# High expression of miR-125b-2 and SNORD116 noncoding RNA clusters characterize ERG-related B cell precursor acute lymphoblastic leukemia

**DOI:** 10.18632/oncotarget.16392

**Published:** 2017-03-21

**Authors:** Elena Vendramini, Marco Giordan, Emanuela Giarin, Barbara Michielotto, Grazia Fazio, Gianni Cazzaniga, Andrea Biondi, Daniela Silvestri, Maria Grazia Valsecchi, Martina U. Muckenthaler, Andreas E. Kulozik, Valter Gattei, Shai Izraeli, Giuseppe Basso, Geertruy te Kronnie

**Affiliations:** ^1^ Department of Women's and Children's Health, University of Padova, Padova, Italy; ^2^ Edmond and Lily Safra Children's Hospital, Sheba Medical Center, Ramat Gan, Israel; ^3^ Tel Aviv University, Tel Aviv, Israel; ^4^ Centro Ricerca Tettamanti, Clinica Pediatrica, University of Milano-Bicocca, Monza, Italy; ^5^ School of Medicine and Surgery, University of Milano-Bicocca, Milano, Italy; ^6^ Department of Pediatric Oncology Hematology, University of Heidelberg, Heidelberg, Germany; ^7^ Clinical and Experimental Onco-Hematology Unit, Centro di Riferimento Oncologico, I.R.C.C.S., Aviano (PN), Italy

**Keywords:** B cell precursor acute lymphoblastic leukemia, ERG aberrations, noncoding RNAs, miR-125, SNORD116

## Abstract

ERG-related leukemia is a B cell precursor acute lymphoblastic leukemia (BCP ALL) subtype characterized by aberrant expression of DUX4 and ERG transcription factors, and highly recurrent *ERG* intragenic deletions. ERG-related patients have remarkably favorable outcome despite a high incidence of inauspicious *IKZF1* aberrations.

We describe clinical and genomic features of the ERG-related cases in an unselected cohort of B-other BCP ALL pediatric patients enrolled in the AIEOP ALL 2000 therapeutic protocol. We report a small noncoding RNA signature specific of ERG-related group, with up-regulation of miR-125b-2 cluster on chromosome 21 and several snoRNAs in the Prader-Willi locus at 15q11.2, including the orphan SNORD116 cluster.

## INTRODUCTION

Childhood B cell precursor acute lymphoblastic leukemia (BCP ALL) is an heterogeneous disease characterized by recurrent genetic aberrations and frequent mutations of genes involved in hematopoietic development, patients that lack common aberrations are defined “B-others” [1].

ERG-related leukemia is a BCP ALL subtype firstly identified by Yeoh and colleagues in the beginning of the gene expression era as a group of B-others characterized by a unique gene expression profile [2]. Recent studies uncovered *IGH-DUX4* rearrangements, and rare *ERG-DUX4* rearrangements, as universal feature and founder event in the ERG-related leukemia subtype [3–6]. In these *DUX4* rearranged leukemias, the double-homeobox transcription factor DUX4 is in most cases placed under the control of the immunoglobulin heavy chain enhancer, is expressed at high level and activates transcription of many genes including a novel ERG (v-ets erythroblastosis virus E26 oncogene homolog) isoform called “ERGalt”. ERGalt protein was proven to inhibit wild-type ERG transcriptional activity and to transform hematopoietic precursors [6].

High frequency of focal mono-allelic *ERG* intragenic deletions were exclusively identified in ERG-related patients [7], the deletion was shown to have subclonal nature and to be caused by aberrant RAG (recombination activating gene) mediated recombination [8, 9]. ERG-related patients were associated to a favorable outcome [10] also despite a marked incidence of *IKZF1* aberrations, a known unfavorable prognostic marker in BCP ALL [8, 9].

So far, very little is known about noncoding RNAs (ncRNAs) expression in this leukemia subtype. Expression of a long noncoding RNA proximal to the first exon of *ERG*, termed “Antisense long noncoding RNA associated with ERG (or ALE)”, was described in the majority of ERG-related cases with *IGH-DUX4* rearrangements and confined to this leukemia subtype. ALE transcripts were shown to be retained at the *ERG* locus in the nucleus and their function has still to be uncovered [6].

Here, we investigated the expression of small noncoding RNAs in ERG-related patients identified among a cohort of B-other BCP ALL enrolled in the AIEOP ALL 2000 therapeutic protocol. We focused on microRNA (miRNAs), protein post-transcriptional regulators, and small nucleolar RNAs (snoRNAs), conserved nuclear RNAs that guide post-transcriptional modification of ribosomal RNAs, and small nuclear RNAs. For the first time we report a specific noncoding RNA signature of ERG-related patients, characterized by high expression of miRNAs in the miR-125b-2 cluster and a subgroup of snoRNAs mapping in the Prader-Willi Syndrome locus.

## RESULTS

### Study cohort

We studied 143 “B-others” specimens at diagnosis of BCP ALL. Patients were enrolled in the AIEOP-BFM ALL 2000 study and lacked genomic aberrations (t(9;22), t(12;21), t(1;19), t(4;11)) or a hyperdiploid karyotype (DNA index >1.16). Patients were mostly younger than ten years (76.9%), had non-high risk MRD levels at day 78 (72,8%) and were assigned to the non-high risk protocol strata (76.9%). High WBC at diagnosis and dismal early treatment response were over-represented in the study group (Supplementary Table 1). Nevertheless, event-free survival (EFS) and cumulative relapse incidence (CRI) analysis revealed no significant differences between B-others included and not included in the study (Supplementary Figure 1). Overall, patients in the study cohort can be considered representative for B-others enrolled in the AIEOP ALL 2000 protocol.

### Highly specific and sensitive classification of patients with an ERG-related signature among B-other BCP ALL

**Figure 1:**
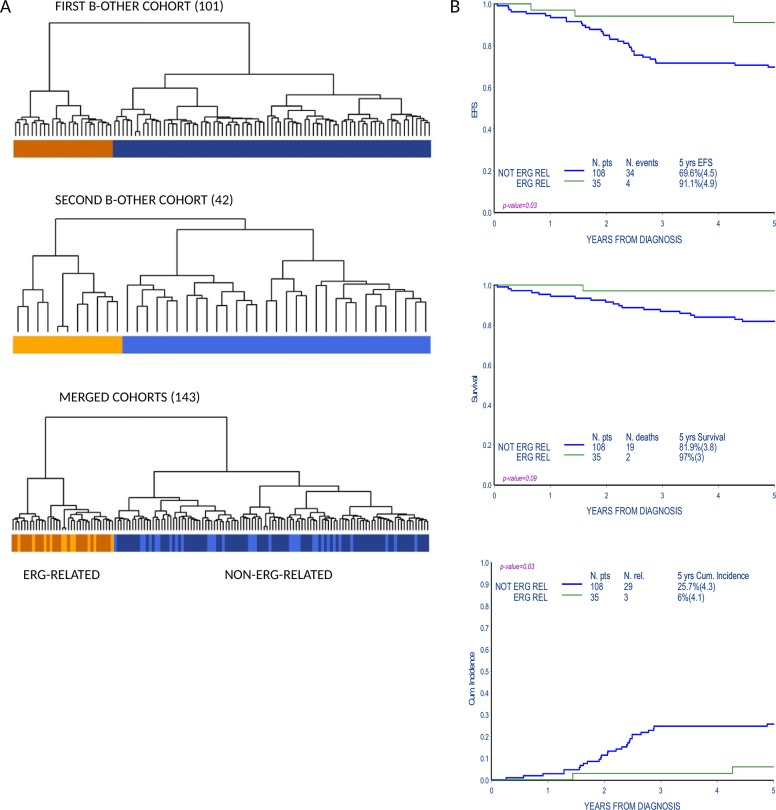
Gene expression profile analysis identifies a subgroup of B-others with favorable outcome **(A)** Unsupervised cluster analysis of B-other cohorts according to gene expression profiles (101 patients in the first cohort, 42 patients in the second cohort and 143 patients in the merged cohort). Groups that cluster apart in the first (brownish, top panel) and second (orange, middle panel) cohort respectively, cluster together when the two cohorts are merged and reanalyzed (lower panel). Patients in the orange/brownish cluster are referred in the paper as ERG-related patients. **(B)** Event free survival (EFS), overall survival (OS) and cumulative incidence of relapse (CIR) of patients in the B-other cohort according to the distinction in unsupervised gene expression analyses: ERG-related and non-ERG-related.

Gene expression profile (GEP) analysis of an initial set of B-others (101 patients) identified a subgroup of patients that clustered separately from the rest of the cohort by unsupervised hierarchical cluster analysis. The same result was obtained in a second independent set of patients (42 patients) analyzed with the purpose to enlarge the study cohort. Twenty-four patients from the first cohort (23.8%) and 11 patients from the second cohort (26.2%) belonged to this tightly clustered subgroup, for a total of 35 out of 143 patients in the merged cohort (Figure 1A).

GEP data of the merged cohort were explored to uncover the features of the tightly clustered patients and class comparison analysis generated a gene lists of 1323 probe sets differently expressed compared to the remaining B-other patients (Supplementary Table 2).

Compared to previously reported BCP ALL subgroups our signature shared 86/100 probe sets with patients described by Harvey et al. [10] as “Cluster 6” in a cohort of high risk ALL (St. Jude cohort, Children's Oncology Group) and representing the ERG-related group in that cohort (Supplementary Figure 2).

ERG-related BCP ALL, classified by GEP, have a prevalence of 24.5% (35 out 143) among the AIEOP B-others cohort with a higher frequency of female patients (p-value 0.02) and lower WBC (p-value 0.004). Other clinical parameters were not significantly different in ERG-related vs. non-ERG-related patients (Table 1).

**Table 1:** Clinical features of 143 patients of this study

	Non-ERG-related		ERG-related		ERG-related without *ERG* deletion		ERG-related with *ERG* deletion	
	N	%	N	%	N	%	N	%
**Total n. of patients**	108		35		20		14	
**GENDER**								
Male	65	60.2	13	37.1	7	35.0	5	35.7
Female	43	39.8	22	62.9	13	65.0	9	64.3
*p-value=0.02*								
**AGE**								
1-5 yrs	60	55.6	13	37.1	7	35.0	5	35.7
6-9 yrs	24	22.2	13	37.1	7	35.0	6	42.9
10-17 yrs	24	22.2	9	25.8	6	30.0	3	21.4
*p-value=0.12*								
**WBC**								
<20000	48	45.3	27	77.1	13	65.0	13	92.9
20-100000	41	38.7	7	20.0	6	30.0	1	7.1
≥100000	17	16.0	1	2.9	1	5.0	0	
Not known	2		0					
*p-value=0.004*								
**PREDNISONE RESPONSE**								
Good	89	84.0	29	82.9	16	80.0	13	92.9
Poor	17	16.0	6	17.1	4	20.0	1	7.1
Not known	2		0					
*p-value (NK excluded)=0.88*								
**MRD STRATIFICATION**								
Standard	31	28.7	5	14.3	3	15.0	2	14.3
Medium	47	43.5	21	60.0	11	55.0	10	71.4
High	12	11.1	5	14.3	3	15.0	1	7.1
Not known	18	16.7	4	11.4	3	15.0	1	7.1
*p-value (NK excluded)=0.16*								
**FINAL RISK**								
Standard	29	26.8	5	14.3	3	15.0	2	14.3
Medium	54	50.0	22	62.8	11	55.0	11	78.6
High	25	23.2	8	22.9	6	30.0	1	7.1
*p-value=0.28*								

To the left, features of 143 patients in the B-other cohort according to the distinction in unsupervised gene expression analyses: ERG-related, non-ERG-related. To the right, features of 34 out of 35 ERG-related patients according to the status of the *ERG* intragenic deletion (one ERG-related patient not analyzed for the *ERG* intragenic deletion was excluded). Clinical parameters were not evaluated by statistical tests because of low patients numerosity. WBC, white blood cells; yrs, years; MRD, minimal residual diseases.

ERG-related group showed a remarkably favorable response to therapy, indeed the cumulative relapse incidence of ERG-related patients was significantly lower compared to the rest of the cohort (5-year CRI: 6%±4.1 vs. 25.7%±4.3, p-value 0.03; 3/35 vs. 29/108 relapses, respectively); accordingly event free survival was significantly higher in ERG-related patients (5-year EFS: 91.1%±4.9 vs. 69.6%±4.5, p-value 0.03; 4/35 vs. 34/108 events, respectively) (Figure 1B and Supplementary Table 3).

Considering the remarkably favorable outcome of the ERG-related patients in our and previous studies a classifier was constructed applying LASSO [11] on all probe sets to improve identification of ERG-related patients. A 91 probe sets classifier was identified (Supplementary Table 4) and was further assessed for discriminative ability using the misclassification rate as performance measure. Considering the outer loop cross validation, a misclassification rate of 0.0062 (sensitivity 0.98, and specificity 1) was obtained. Applying this classifier to the study cohort, all patients were correctly identified as either ERG-related or non-ERG-related.

### Incidence of ERG aberrations in ERG-related BCP ALL

*ERG* intragenic deletions were early on described in ERG-related leukemia and mostly involve conserved breakpoints between exon2 and exon10 of *ERG* isoform 1 (NM_182918) [8, 9].

In our study long-template PCR established to detect deletions between exon2 and exon10 of *ERG* identified intragenic deletions in 11 out of 27 ERG-related samples and in none of non-ERG-related patients, genomic breakpoints were characterized in 8/11 samples. As reported previously [8, 9] breakpoints downstream exon2 were very consistent among samples whereas the breakpoints upstream exon10 were located within a 2.5kb genomic region. In 4 samples co-occurrence of multiple breakpoints was identified (Supplementary Figure 3 and Supplementary Table 5). Most recent data uncovered less common deletion patterns that involve the first exon or the whole gene *ERG* [6], and might have been missed in our analysis.

To further assess *ERG* aberrations in our cohort, the presence of *ERG* deleted transcripts was investigated on cDNA of 31 ERG-related and 84 non-ERG-related patients. Expression of *ERG* deleted transcripts revealing the presence of the genomic deletions was found in 14 (including the 11 samples identified by gPCR) out of 31 ERG-related patients and in none of the non-ERG-related samples. In all deleted samples wild type *ERG* mRNA was detected as well, in line with previous observations that BCP ALL *ERG* intragenic deletions are monoallelic. Altogether, 14 out of 34 (41.2%) investigated ERG-related patients harbor *ERG* intragenic deletions.

### Similarities and differences within ERG-related specimens: ERG deleted and ERG non-deleted leukemias

The presence of *ERG* intragenic deletions in about half of ERG-related patients raises the question if the latter can be considered a unique homogeneous group.

*ERG-wt* gene expression level was analyzed by qRT-PCR (probe *ERG-wt* specific) in 31 ERG-related and 71 non-ERG-related B-others samples. Surprisingly, no difference in *ERG-wt* expression was found among ERG-related samples with and without *ERG* intragenic deletions. *ERG-wt* expression was highly heterogeneous in non-ERG-related samples and modestly higher when compared to ERG-related specimens, with a less than two-fold difference between the means (0.9916 ± 0.1176 relative expression in ERG-related vs. 1.789 ± 0.2513 in non-ERG-related; t-test, p-value=0.005; Supplementary Figure 4A-4B) in keeping with previous observations [12]. Western blotting analysis in a representative subgroup of patients (13 ERG-related and 4 non-ERG-related) revealed similar levels of ERG protein expression in all samples (data not shown). An additional short ERG isoform, supposedly the ERGalt protein described in most of ERG-related patients and shown to inhibit wild type ERG transcriptional activity [6], was expressed in 6 out of 13 ERG-related patients (46,1%; 2/5 with *ERG* deletion and 4/8 without *ERG* deletion) and in none of non-ERG-related cases (Supplementary Figure 4C).

Wondering if inhibition of ERG transcriptional program was a general event in ERG-related patients, we applied GSEA analysis to our gene expression data. Consistently, a signature of ERG-activated genes from literature [13] was significantly enriched in non-ERG-related patients when compared to ERG-related group (Supplementary Figure 5) suggesting an impaired ERG signaling in the ERG-related leukemia subtype.

We further investigated if global gene expression would distinguish *ERG*-deleted patients within the ERG-related group. Unsupervised hierarchical cluster analysis of ERG-related expression profiles revealed a different gene expression signature among the samples with and without a deletion, indeed the two groups clustered separately (Supplementary Figure 6A). T-test analysis revealed a 232 probe sets signature (t-test, p<0.05, Supplementary Table 6).

Interestingly, among the differentially regulated genes we found a significant higher expression of *RAG-1* and *RAG-2* in *ERG* deleted compared to *ERG* not deleted samples (Supplementary Figure 6B-6C). This supports the hypothesis raised in previous studies [8, 9] that *ERG* intragenic deletion takes place by RAG-mediated rearrangement.

We conclude that while ERG-related leukemias share most clinical features and can be considered a unique group, some biological differences between *ERG* deleted and *ERG* not deleted leukemias are noteworthy.

### ERG-related status influences the incidence of genomic aberrations and outcome

**Figure 2:**
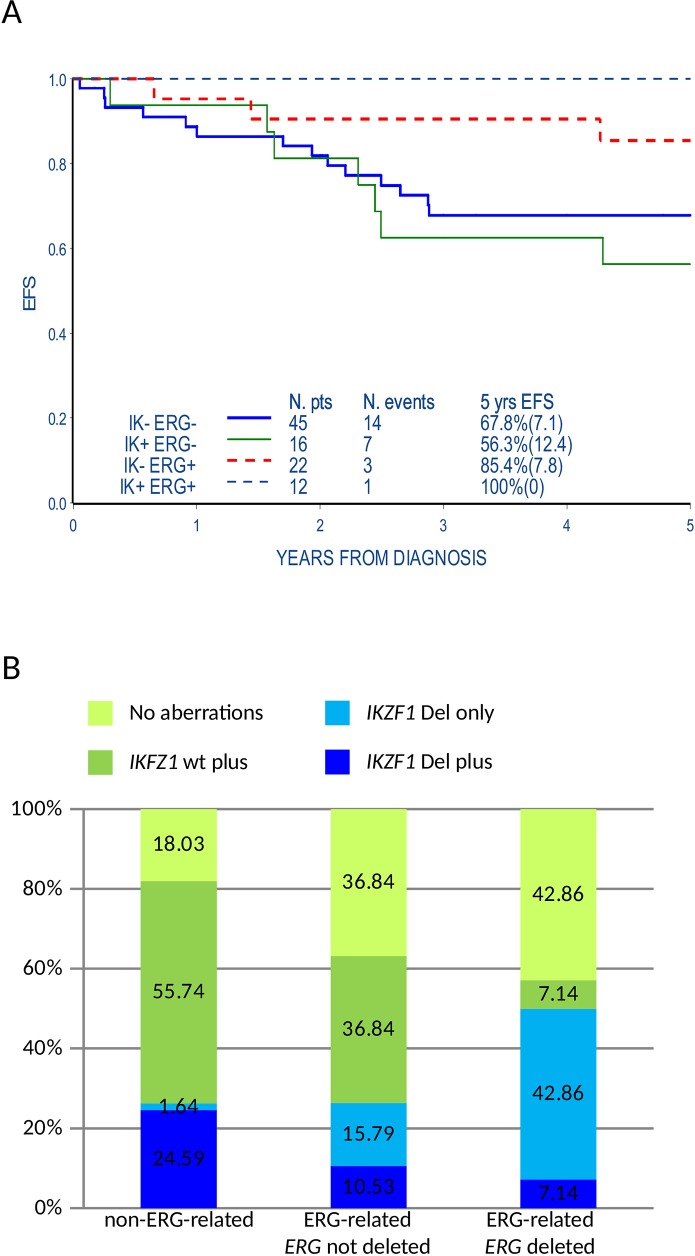
ERG-related status influences the incidence of genomic microdeletions and their association with prognosis **(A)** Event free survival (EFS) of ERG-related and non-ERG-related B-others patients analyzed by MLPA (95) according to the presence of *IKZF1* aberrations. Events occurred later than 5 years from diagnosis are also indicated in the figure but are not included in the EFS curve (e.g. IK+ERG+ annotated event occurred 6.9 years from diagnosis). IK+: presence of *IKZF1* aberrations (intragenic or whole gene); IK-: absence of *IKZF1* aberrations; ERG+: ERG-related; ERG-: non-ERG-related. **(B)** Histogram representing the incidence of *IKZF1* aberrations isolated or co-occurring with the other genomic microdeletions included in the SALSA MLPA P335-B1 ALL-IKZF1 assay in ERG-related patients with or without *ERG* intragenic deletion and in non-ERG-related B-other patients (95). *IKZF1* Del plus: *IKZF1* aberrations (intragenic or whole gene deletions) and one or more aberrations of B-cell differentiation genes*; IKZF1* Del only: *IKZF1* aberrations in the absence of aberrations of B-cell differentiation genes; *IKZF1* wt plus: lack of *IKZF1* aberrations but aberrations of B-cell differentiation genes ; No aberrations of B-cell differentiation genes including *IKZF1*.

Samples expressing the ERG-related signature lacked major chromosomal aberrations frequently found in BCP ALL. We questioned if the presence of the ERG-related mRNA signature was associated with the incidence of genomic micro-deletions of B cell differentiation pathway genes [14]. Eight recurrent aberration sites (*IKZF1, P2RY8-CRLF2, CDKN2A/B, PAX5, ETV6, BTG1, RB1, EBF1*) were analyzed by MLPA in 95/143 patients of the B-others cohort (34 ERG-related, among them 14 *ERG* deleted, and 61 non-ERG-related samples). When compared to the non-ERG-related samples, ERG-related samples have a statistically significant lower incidence of aberrations involving the *CDKN2A/B* and *PAX5* (49,2% vs. 14.7% and 47.4% vs. 11.8% respectively, p<0.001) while we found only a trend of lower frequency of *P2RY8-CRLF2, ETV6, BTG1*, *RB1* and *EBF1* aberrations (Table 2). Conversely, a slightly higher frequency of *IKFZ1* aberrations was found in the ERG-related patients (35.3% of ERG-related vs. 26,2% of non-ERG-related, p-value=0.35). The difference was more evident considering *ERG* deleted patients compared to the rest of the cohort (50% vs. 26,25%, p-value=0.07), in line with previously reported data [8, 9]. The presence of *IKZF1* aberrations did not affect the highly favorable prognosis of ERG-related patients, both *ERG* deleted and not deleted, while it was associated to a worst EFS at 5 years from diagnosis in the non-ERG-related patients (5-year EFS: 100% vs. 56,3%±12.4, p-value=0.04) (Figure 2A). Beside the overall higher incidence of *IKZF1* aberrations, we noticed in ERG-related patients, both *ERG* deleted and not deleted, that *IKZF1* aberrations occur mainly as single aberrations (*IKZF1* only) while among the non-ERG-related patients *IKZF1* aberrations co-occurred with ≥1 aberrations analyzed by MLPA *(IKZF1* plus) (3 patients *IKZF1* plus and 9 *IKZF1* only in ERG-related vs. 15 *IKZF1* plus and 1 *IKZF1* only in non-ERG-related, p-value=0.0002; Figure 2B and Supplementary Table 7).

**Table 2:** Incidence of genomic aberrations analyzed by SALSA MLPA P335-B1 ALL-IKZF1

	Non-ERG-related		ERG-related		ERG-related without *ERG* deletion		ERG-related with *ERG* deletion	
	N	%	N	%	N	%	N	%
**Total n. of patients**	108		35		20		14	
***IKZF1***								
Not deleted	45	73.8	22	64.7	14	73.7	7	50.0
Deleted	16	26.2	12	35.3	5	26.3	7	50.0
Not known	47		1		1		0	
*p-value (NK excluded)=0.35*								
***P2RY8-CRLF2***								
Not deleted	52	85.2	32	94.1	18	94.7	13	92.9
Deleted	9	14.8	2	5.9	1	5.3	1	7.1
Not known	47		1		1		0	
*p-value (NK excluded)=0.32*								
***CDKN2A/B***								
Not deleted	31	50.8	29	85.3	14	73.7	14	100.0
Deleted	30	49.2	5	14.7	5	26.3	0	
Not known	47		1		1		0	
*p-value (NK excluded)<0.001*								
***PAX5***								
Not deleted	32	52.6	30	88.2	16	84.2	13	92.9
Deleted	29	47.4	4	11.8	3	15.8	1	7.1
Not known	47		1		1		0	
*p-value (NK excluded)<0.001*								
***ETV6***								
Not deleted	50	82.0	31	91.2	16	84.2	14	100.0
Deleted	11	18.0	3	8.8	3	15.8	0	
Not known	47		1		1		0	
*p-value (NK excluded)=0.37*								
***BTG1***								
Not deleted	55	90.2	33	97.1	19	100.0	13	92.9
Deleted	6	9.8	1	2.9	0		1	7.1
Not known	47		1		1		0	
*p-value (NK excluded)=0.42*								
***RB1***								
Not deleted	55	90.2	32	94.1	17	89.5	14	100.0
Deleted	6	9.8	2	5.9	2	10.5	0	
NK	47		1		1		0	
*p-value (NK excluded)=0.71*								
***EBF1***								
Not deleted	56	91.8	34	100.0	19	100.0	14	100.0
Deleted	5	8.2	0		0		0	
Not known	47		1		1		0	
*p-value (NK excluded)=0.16*								

To the left, incidence of aberrations in 95/143 patients in the B-others cohort according to the distinction in unsupervised gene expression analyses: ERG-related (34/35), non-ERG-related (61/108). To the right, incidence of aberrations in 33 out of 34 ERG-related patients according to the presence of *ERG* intragenic deletion (one ERG-related patients not analyzed for the *ERG* intragenic deletion was excluded). Clinical parameters were not evaluated by statistical tests because of low patients numerosity.

### ERG related BCP ALL patients share a microRNA signature with overexpression of the miR-125b-2 cluster

Considering the highly distinct gene expression program that distinguishes ERG-related from non-ERG-related patients, further analysis including other RNA species was pursued.

**Figure 3:**
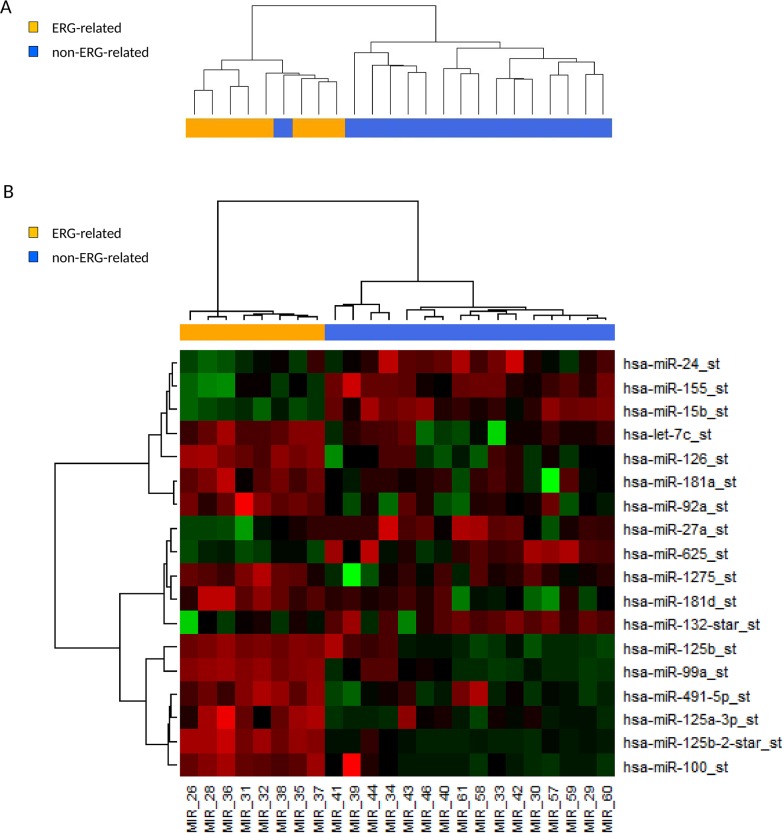
ERG-related BCP ALL share a unique microRNA expression signature with high expression of the miR-125b-2 cluster **(A)** Dendrogram of unsupervised hierarchical clustering of 24 B-others patients on 847 human miRNAs probe sets of the microRNA 1.0 Affymetrix microarray. ERG-related patients (8) cluster apart from the non-ERG-related patients (16). **(B)** Heatmap of 18 differentially regulated microRNAs between 8 ERG-related and 16 non-ERG-related B-others patients identified by class comparison analysis (adjusted p-value <0.05).

Noncoding RNAs expression profile analysis was performed on 24 BCP ALL patients of the study cohort, 8 ERG-related and 16 non-ERG-related B-others patients. Unsupervised cluster analysis clustered the ERG-related patients separately from the non-ERG-related patients (Figure 3A). Class comparison analysis (Wilcoxon test) resulted in a signature of 18 differentially regulated miRNAs (Table 3 and Figure 3B). Top-ranked in the signature were all miRNAs belonging to miR-125b-2 cluster (hsa-miR-125b, -125b-2*, -99a, let-7c). Overexpression in ERG-related patients was validated by qRT-PCR in a second group of 40 patients, 20 ERG-related and 20 non-ERG-related, belonging to the initial study cohort (Supplementary Figure 7).

**Table 3:** MicroRNAs probe sets differentially expressed between ERG-related and non-ERG-related patients identified by class comparison analysis (18 probe sets, adjusted p-value <0.05)

Probe set	Chr locus	p-value	means ERG-related	means non-ERG-related	FC (mean ERG-related/mean non-ERG-related)
hsa-miR-125b-2-star_st	21q21.1	0.002	5.126	0.943	5.4371
hsa-miR-99a_st	21q21.1	0.002	8.819	2.156	4.0911
hsa-miR-100_st	11q24.1	0.017	4.440	1.387	3.2015
hsa-miR-125b_st	21q21.1; 11q24.1	0.015	9.926	3.845	2.5814
hsa-miR-125a-3p_st	19q13.41	0.016	3.506	1.500	2.3381
hsa-miR-491-5p_st	9p21.3	0.031	3.589	1.592	2.2548
hsa-miR-126_st	9q34.3	0.002	10.025	6.981	1.4360
hsa-miR-1275_st	6p21.31	0.035	7.449	6.046	1.2322
hsa-miR-181d_st	19p13.13	0.022	7.056	5.975	1.1809
hsa-let-7c_st	21q21.1	0.029	11.243	9.729	1.1555
hsa-miR-181a_st	1q32.1; 9q33.3	0.035	12.940	12.454	1.0391
hsa-miR-92a_st	13q31.3; Xq26.2	0.015	12.466	12.063	1.0334
hsa-miR-24_st	9q22.33; 19p13.13	0.035	9.442	10.234	0.9225
hsa-miR-15b_st	3q25.33	0.002	8.183	9.006	0.9086
hsa-miR-155_st	21q21.3	0.003	8.695	10.239	0.8492
hsa-miR-27a_st	19p13.13	0.035	5.789	7.061	0.8198
hsa-miR-625_st	14q23.3	0.022	5.550	7.016	0.7910
hsa-miR-132-star_st	17p13.3	0.035	3.720	5.710	0.6516

Furthermore, hsa-miR-100 belonging to the miR-125b-1 cluster (chr11, *MIR100HG* host gene) and hsa-miR-125a-3p belonging to miR-125a cluster (chr19) were among the top ranked differentially expressed miRNAs, highlighting the involvement of the miR-125 family in the ERG-related leukemia phenotype.

### Higher expression of snoRNAs in Prader-Willi Syndrome locus in ERG-related BCP ALL

**Figure 4:**
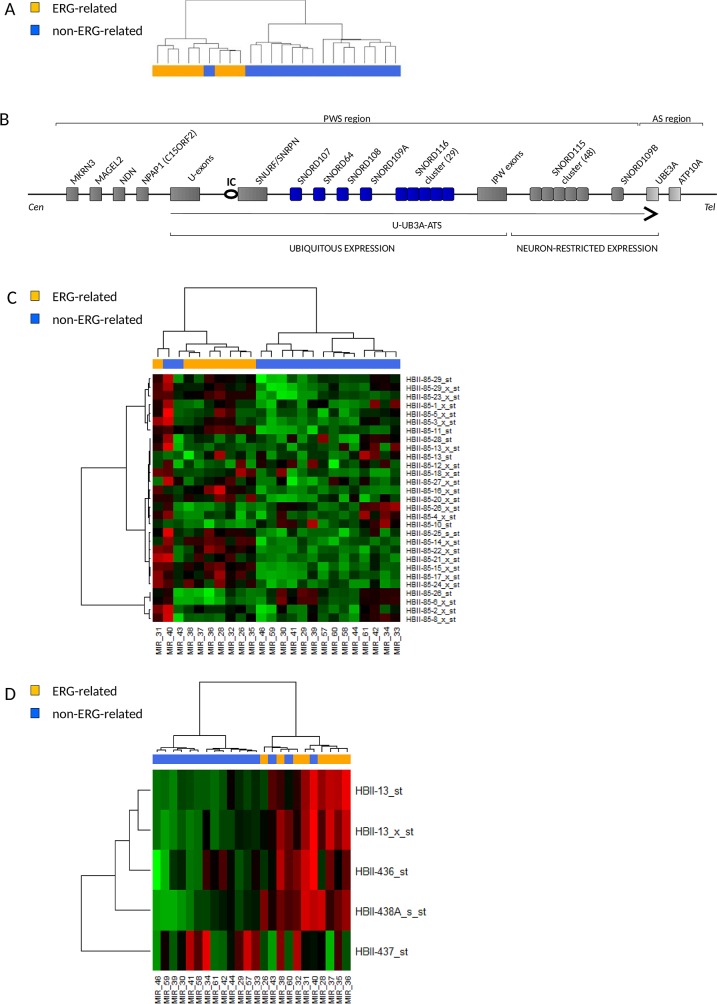
ERG-related BCP ALL share a unique short noncoding RNA expression signature with high expression of snoRNAs in the Prader-Willi Syndrome locus **(A)** Dendrogram showing unsupervised hierarchical clustering of 24 B-others patients on 922 noncoding RNAs probe sets of the microRNA 1.0 Affymetrix microarray. ERG-related patients (8) cluster apart from the non-ERG-related patients (16). **(B)** Schematic representation of the imprinted human chromosomal locus 15q11-q13 that hosts the Prader-Willi Syndrome (PWS) and Angelman Syndrome (AS) regions. Paternally expressed genes are shown with dark grey while maternally expressed genes are shown in light grey. A circle indicates the PWS imprinting center (IC) and the U-UBE3A-ATS transcript is shown as a black arrow. SnoRNAs differentially expressed in ERG-related leukemia are highlighted in blue. Representation is not to scale. **(C-D)** Heatmaps of the expression of snoRNAs in the PWS locus in 8 ERG-related and 16 non-ERG-related B-other patients. **(C)** Expression of the probes in the SNORD116 (HBII-85) cluster. **(D)** Expressions of SNORD64 (HBII-13), SNORD107 (HBII-436), SNORD108 (HBII-437) and SNORD109A (HBII-438A) are shown. Probes in the SNORD115 cluster, known to be neuron-specific [17], were not expressed in none of the samples (data not shown) whereas SNORD109B (neuron-specific), SNORD116-7, -9, -19, -30 were not represented by probe sets of the microRNA 1.0 Affymetrix microarray.

Unsupervised cluster analysis on small nucleolar RNAs (snoRNAs) probe sets again showed tight clustering of ERG-related patients (Figure 4A). Class comparison analysis detected a list of differentially expressed probe sets (Table 4) most of which mapping in the 15q11-q13 chromosomal region, a locus governed by genomic imprinting and involved in the neurodevelopmental congenital disease Prader-Willi Syndrome (PWS) [15, 16] (Figure 4B).

**Table 4:** Small Nucleolar RNAs probe sets differentially expressed between ERG-related and non-ERG-related patients identified by class comparison analysis

Probe set	Chr locus	Gene name	snoRNA type	p-value	means ERG-related	means non-ERG-related	FC (mean ERG-related/mean non-ERG-related)
HBII-85-16_x_st	15q11.2	SNORD116-16	C/D box	0.002	1.343	0.422	3.1847
HBII-85-22_x_st	15q11.2	SNORD116-22	C/D box	0.004	1.667	0.603	2.7627
HBII-85-17_x_st	15q11.2	SNORD116-17	C/D box	0.002	1.883	0.753	2.5000
HBII-85-11_st	15q11.2	SNORD116-11	C/D box	0.004	3.253	1.353	2.4047
HBII-85-15_x_st	15q11.2	SNORD116-15	C/D box	0.003	1.995	0.837	2.3832
HBII-85-24_x_st	15q11.2	SNORD116-24	C/D box	0.01	1.7	0.786	2.1641
HBII-85-14_x_st	15q11.2	SNORD116-14	C/D box	0.004	1.498	0.709	2.1123
HBII-85-23_x_st	15q11.2	SNORD116-23	C/D box	0.003	4.491	2.243	2.0022
HBII-438A_s_st	15q11.2	SNORD109A	C/D box	0.004	3.887	1.961	1.9819
HBII-85-21_x_st	15q11.2	SNORD116-21	C/D box	0.012	1.418	0.717	1.9773
HBII-13_st	15q11.2	SNORD64	C/D box	0.016	2.955	1.626	1.8171
HBII-13_x_st	15q11.2	SNORD64	C/D box	0.013	2.673	1.5	1.7818
ENSG00000212326_st	2p32.2	ENSG00000212326		0.004	1.557	0.875	1.7789
U47_st	1q25.1	SNORD47	C/D box	0.03	1.224	0.707	1.7326
mgU6-77_st	17p13.1	SNOR10	C/D box	0.004	1.552	0.909	1.7072
HBII-296B_st	17p13.3	SNORD91B	C/D box	0.017	1.083	0.64	1.6926
ACA17_st	9q34.3	SNORA17	H/ACA box	0.003	2.762	1.699	1.6259
ENSG00000201199_s_st	11q21	SCARNA9		0.035	1.386	0.869	1.5956
U53_st	2p23.2	SNORA53	C/D box	0.002	4.007	2.573	1.5574
U35B_st	19q13.33	SNORD35B	C/D box	0.002	2.382	1.556	1.5309
HBII-85-10_st	15q11.2	SNORD116-10	C/D box	0.013	0.611	1.088	0.5620

Deregulated probe sets with fold change >1.5 or fold change <1/1.5 (adjusted p-value <0.05) are listed (probe sets identified by comparison analysis but with mean intensity <1 in both groups were excluded from the list).

The PWS locus hosts six C/D box snoRNA species, among them SNORD64 (HBII-13), SNORD107 (HBII-436), SNORD108 (HBII-437), SNORD109A (HBII-438A) and SNORD116 (HBII-85) are reported to be ubiquitously expressed, whereas SNORD109B (HBII-438B) and SNORD115 (HBII-52) are restricted to expression in neurons [17].

The analysis revealed a specific up-regulation of SNORD109A, SNORD64, SNORD107 and 12 snoRNAs in the SNORD116 cluster (SNORD116-11, -14, -15, -16, -17, -18, -20, -21, -22, -23, -24, -27) in ERG-related patients (Figure 4C–4D and Supplementary Figure 8).

The SNORD116 cluster is organized in a large tandemly repeated cluster containing 29 gene copies of snoRNAs with highly similar sequences. Based on sequence similarity, members of the SNORD116 cluster were proposed to be divided into three snoRNA groups (SNOG). SNOG1 (SNORD116-1 to -9) was reported to have significantly higher expression than SNOG2 (SNORD116-10 to -24) or SNOG3 (SNORD116-25 to-29) in several tissues [18].

Interestingly, most of SNORD116 snoRNAs up-regulated in ERG-related samples belong to SNOG2 and share high similar anti-sense elements (ASEs), the sequences up-stream to D and D’ box that guide the snoRNA to the target by complementarity (Supplementary Table 8).

## DISCUSSION

This study shows that a specific ERG-related GEP signature identifies 25% of patients among BCP ALL B-other patients lacking t(4;11), t(12;21), t(9;22), t(1;19) and with 1.16>DNA index <1.60. The study cohort of 143 B-other patients reported here includes all risk groups and is representative of B-others in the AIEOP ALL 2000 protocol study offering generality of our findings.

In our study 40% of patients with an ERG-related signature carry *ERG* intragenic deletions involving central exons, rare deletions involving first or last exons recently described [6] might have been missed in our analysis. Deletions were in all cases monoallelic and in many cases showing evidence of subclonal nature. We observed no age related enrichment neither for patients with an ERG-related signatures nor for *ERG* deleted patients. We did observe a sex related increase of ERG-related as well as *ERG* deleted cases being female patients overrepresented in both groups, which is remarkable considering that female patients are underrepresented among B-other patients.

Overall, patients with a common ERG-related signature comprising *ERG* deleted cases are commonly found among patients lacking classical genetic lesions and importantly, identifies patients showing a good response to assigned therapy.

In addition to the lack of major chromosomal alterations, ERG-related patients had a lower incidence of micro-aberrations of genes related to B cell differentiation known to be frequently deleted or amplified in BCP ALL [14]. Aberrations of *IKFZ1* represent a relevant exception to this trend with a very high incidence (50%) in ERG-related patients carrying intragenic *ERG* deletion. In ERG-related patients *IKZF1* aberrations are mostly found as isolated aberrations not associated with poor prognosis. This observation tempted to speculate that co-occurrence of additional aberrations supports the unfavorable prognosis of *IKZF1* aberrations in non-ERG-related BCP ALL patients, and to reconsider the role of *IKZF1* aberrations alone as poor prognostic marker. Though, another speculation is that *ERG* aberrations are dominant in phenotypic appearance over *IKZF1* deletions. Importantly, IKZF1 was recently identified among *ERG* transcriptional regulators [19], suggesting that *IKZF1* and *ERG* aberrations tightly interrelate in affecting patient's outcome.

We did find down-regulation of a set of ERG downstream target genes in ERG-related patients, suggesting impaired ERG signaling in these samples. ERG-wt expression was not altered in relation to ERG status but 46% of ERG-related patients expressed a short ERG isoform, supposedly the ERGalt protein recently described in another ERG-related cohort [6] and shown to inhibit wild type ERG transcriptional activity.

Strikingly, we show that among ERG-related patients the expression of a set of genes distinguishes patients with and without *ERG* intragenic deletions.

Patients with *ERG* intragenic deletions showed higher expression of recombination activating genes *RAG1* and *RAG2*. The latter offers rational for the enrichment of *ERG* and *IKZF1* intragenic deletions caused by illegitimate RAG-mediated recombination [20, 21]. However, the higher *RAG1/RAG2* expression cannot be considered the only cause of specific *ERG* and *IKZF1* deletion accumulation, indeed other microdeletions mediated by illegitimate RAG-meditated recombination like *BTG1, ETV6, CDKN2A/B* [22] and the *P2RY8/CRLF2* deletion [23] had a very low incidence in ERG-related patients. Presumably additional factors guide the propensity of specific aberrations to occur e.g. active chromatin H3K4me3 was shown to tether the RAG enzyme complex to DNA and to increase RAG enzymatic activity [24].

Besides a unique GEP we show for the first time that ERG-related leukemia share a highly specific noncoding RNA signature. The most deregulated microRNAs belong to the miR-125 family and to the genomic clusters in which they are embedded. Indeed, the clusters miR-125b-2 and miR-125b-1 were shown to be transcribed as a single polycistronic RNA from their host genes *LINC00478* and *MIR100HG* respectively in normal and leukemic hematopoietic cells [25]. All three guide-strands of the miR-125 family share the same seed region and were shown to have the same biological phenotype when overexpressed in murine cells [26]. The miR-125 family considered as ‘oncomir’[27] has been reported to play a fundamental role in hematopoiesis, is highly expressed in several leukemia subtypes [28] and involved in rare chromosomal translocations in ALL [29] with transformation of hematopoietic progenitors and repression of pro-apoptotic genes.

In addition to a miRNA signature, ERG-related patients cluster apart from other BCP ALL patients with a specific snoRNA expression profile. Strikingly, the most differentially regulated snoRNAs map to the 15q11.2 chromosomal region governed by genomic imprinting and are involved in the Prader-Willi Syndrome (PWS). A subgroup of snoRNAs in the PWS locus, including the SNORD116 cluster, was specifically up-regulated in ERG-related patients whereas others were heterogeneously expressed in the cohort. With the exceptions of SNORD115, which has been shown to regulate the alternative splicing of the 5HT-2C serotonin receptor pre-mRNA [30], all other snoRNAs in the PWS locus are “orphan snoRNAs” that lack any complementarity with known ribosomal RNA or small nuclear RNA and their non-canonical function is mostly unknown [31].

Up-regulation of snoRNAs in the SNORD116 cluster were previously described in a subgroup of multiple myeloma patients where expression levels were associated to genome-wide copy number alterations in the genomic locus [32]. Specific patterns of snoRNA expression were described in cytogenetic subgroups of chronic lymphocytic leukemia, and in particular high SNORD116-18 expression was associated to shorter progression free survival [33]. Here for the first time we describe the up-regulation of snoRNAs in PWS locus associated to a favorable outcome in childhood BCP ALL. Interestingly, SNORD116 snoRNAs that are up-regulated in ERG-related patients share high similar anti-sense elements (ASEs) suggesting a common biological function.

All together our data contribute to the knowledge on the recently defined ERG-related leukemia subtype, that despite the presence of *IKZF1* deletions are marked by an excellent prognosis. A specific small noncoding RNA signature shared by ERG-related patients is presented and offers new clues to dissect their specific biological program.

## MATERIALS AND METHODS

### Patients’ cohort

A cohort of 143 pediatric patients with diagnosis of B cell precursor acute lymphoblastic leukemia (BCP ALL) were included in the study. Patients were routinely tested for recurrent genomic aberrations (t(9;22)/*BCR-ABL*, t(12;21)/*TEL-AML1*, t(4;11)/*MLL-AF4*) and DNA index of blast cells and enrolled in the AIEOP-BFM ALL 2000 therapeutic protocol in Italian centers [34]. “B-others” were defined as patients with diagnosis of BCP ALL lacking recurrent genomic aberrations (t(9;22)/*BCR-ABL*, t(12;21)/*TEL-AML1*, t(4;11)/*MLL-AF4*) or a hyperdiploid karyotype (DNA index between 1.16 and 1.6) and not affected by Down syndrome; BCR-ABL1-like cases not excluded. In the study cohort, also patients with t(1;19)/*TCF3-PBX1* were excluded. The local ethics committees approved the study and informed consent was obtained for all patients (NCT00613457).

### RNA and DNA preparation and quantitative analysis

DNA and RNA were isolated from bone marrow or peripheral blood mononuclear cells separated by Ficoll-Hypaque (Pharmacia, Uppsala, Sweden). TaqMan gene expression assay (Applied Biosystems, Foster City, CA, USA) was used to assess *ERG* gene expression (Hs 01554629-m1). MiRNAs expression data were validated by TaqMan^®^ MicroRNA assays (Applied Biosystems). SnoRNAs expression were validated by miScrpt PCR System (Qiagen, Hilden, Germany).

### Genes and ncRNAs expression profiling and data analysis

Gene expression profiles were obtained with HG-U133 Plus 2.0 GeneChip^®^ (Affymetrix, Santa Clara, CA, USA) arrays. A first set of patients (101) was processed as part of the MILE study as previously described [35]. A second set of patients (42) was processed starting from 100ng of total RNA using the GeneChip^®^ 3′IVT express kit and protocol (Affymetrix).

Noncoding RNA expression profiles were obtained with the Mirna array 1.0 GeneChip^®^ (Affymetrix). Total RNA (1μg) was labelled using the FlashTag^TM^ kit (Genisphere, Hatfield, PA) following manufacturer's instructions.

The data discussed in this publication have been deposited in NCBI's Gene Expression Omnibus [36] and are accessible through GEO Series accession number GSE79547 (http://www.ncbi.nlm.nih.gov/geo/query/acc.cgi?acc=GSE79547).

R/Bioconductor packages and Partek software (Partek^®^ Genomics Suite^®^ software, version 6.6 Copyright ©; 2014 Partek Inc., St. Louis, MO, USA) were used for microarrays data analysis. Gene expression profiles of all 143 samples in the study cohort were used to build a classifier and LASSO [11] was used as prediction method. Enrichment of relevant signatures previously published was analyzed using Gene Set Enrichment Analysis (GSEA) software [37, 38].

### Characterization of ERG intragenic deletions

Breakpoints on genomic DNA were investigated by long-range PCR using PCR Extender System (5 Prime, Hilden, Germany). A second PCR was run to better characterize the breakpoints in patients with deletions. Expression of deleted *ERG* transcripts were investigated by PCR on cDNA. PCR products were analyzed by Sanger sequencing.

### Multiplex ligation-dependent Probe Amplification (MLPA) analysis

MLPA analysis has been developed according to the manufacturer's protocol using SALSA MLPA probemix P335-B1 ALL-IKZF1 kit (MRC-Holland, Amsterdam, the Netherlands). For data analysis, Coffalyser.NET version v120309.150 was used.

### Western blot analysis

Whole-cells lysates of leukemia samples at diagnosis were obtained from cryopreserved bone marrow mononuclear cells. Anti-ERG rabbit monoclonal antibody [EPR3864(2)] (ab133264, Abcam) was used to detect ERG proteins, anti-β-actin-HRP (A3854, Sigma) was used as loading control.

### Statistical analysis

Event Free Survival (EFS) and overall survival were estimated according to Kaplan-Meier, with Greenwood standard error and with the log-rank test for comparison; Cumulative Relapse Incidence (CRI) was estimated adjusting for competing risks of other events and compared with the Grey test. To assess associations between patients’ features, the Chi-Square test was applied. GraphPad Prism software and SAS 9.2 were used for analyses (GraphPad Software, La Jolla, CA, USA).

## SUPPLEMENTARY MATERIALS FIGURES AND TABLES














