# Supplementary material for: High expression of miR-125b-2 and SNORD116 noncoding RNA clusters characterize ERG-related B cell precursor acute lymphoblastic leukemia
Source: Oncotarget. 2017 Mar 21;8(26):42398–413. doi: 10.18632/oncotarget.16392 (PMC5522075; doi:10.18632/oncotarget.16392)
Supplement: Supplementary file 1 [file oncotarget-08-42398-s001.pdf]

# High expression of miR-125b-2 and SNORD116 noncoding RNA clusters characterize ERG-related B cell precursor acute lymphoblastic leukemia

## SUPPLEMENTARY MATERIALS AND METHODS

### Patients' cohort

A cohort of 143 pediatric patients with diagnosis of B cell precursor acute lymphoblastic leukemia (BCP ALL) were included in the study. Patients were routinely tested for recurrent genomic aberrations (t(9;22)/*BCR-ABL*, t(12;21)/*TEL-AML1*, t(4;11)/*MLL-AF4*) and DNA index of blast cells and enrolled in the AIEOP-BFM ALL 2000 therapeutic protocol in Italian centers [1]. "B-others" were defined as patients with diagnosis of BCP ALL lacking recurrent genomic aberrations (t(9;22)/*BCR-ABL*, t(12;21)/*TEL-AML1*, t(4;11)/*MLL-AF4*) or a hyperdiploid karyotype (DNA index between 1.16 and 1.6) and not affected by Down syndrome; BCR-ABL1-like cases not excluded. In the study cohort, also patients with t(1;19)/*TCF3-PBX1* were excluded. The local ethics committees approved the study and informed consent was obtained for all patients (NCT00613457).

### RNA and DNA preparation

DNA and RNA were isolated from bone marrow or peripheral blood mononuclear cells separated by Ficoll-Hypaque (Pharmacia, Uppsala, Sweden), extraction were performed either from fresh cells or from stored frozen material. DNA was isolated using the Puregene Cell and Tissue Kit (Qiagen Inc., Valencia, USA). Total RNA were isolated using TRIZOL following manufacturer's instructions (Invitrogen, Paisley, UK), quality control was performed with the 2100 Bioanalyzer using the "Eukaryote total RNA Nano Assay" (Agilent Technologies). All material was stored at -80°C.

### Genes and ncRNAs expression arrays

Gene expression profiles were obtained with HG-U133 Plus 2.0 GeneChip® (Affymetrix, Santa Clara, CA, USA) arrays. A first set of patients (101) was processed as part of the MILE study as previously described [2]. A second set of patients (42) was processed starting from 100ng of total RNA using the GeneChip® 3'IVT express kit and protocol (Affymetrix, Santa Clara, CA, USA).

MiRNA expression profiles were obtained with the Mirna array 1.0 GeneChip® (Affymetrix, Santa Clara, California). This latter array interrogates a total of 7815 probe sets representing miRNAs of 71 organisms (847 human miRNAs) annotated in Mirbase v.11 and 922 human snoRNAs and scaRNAs sequences. Total RNA (1µg) was labelled using the FlashTag™ kit (Genisphere, Hatfield, PA) following manufacturer's instructions.

For both arrays hybridization, staining and washing were performed using protocols as recommended by the manufacturer, stained chip were scanned on GeneChip Scanner 3000 7G (Affymetrix, Santa Clara, California). Expression files (Affymetrix .CEL files) were generated using GeneChip® Operating Software (GCOS) and Affymetrix® GeneChip® Command Console® Software (AGCC) (Affymetrix). The data discussed in this publication have been deposited in NCBI's Gene Expression Omnibus [3] and are accessible through GEO Series accession number GSE79547 (http://www.ncbi.nlm.nih.gov/geo/query/acc.cgi?acc=GSE79547).

### Microarrays data analysis

R/Bioconductor packages and Partek software (Partek® Genomics Suite® software, version 6.6 Copyright ©; 2014 Partek Inc., St. Louis, MO, USA) were used for microarrays data analysis. Arrays have been normalized using robust multiple-array average (RMA) [4]. When data belonged to different protocols batch effects were removed using Combat [5]. Unsupervised analyses were based on hierarchical clustering (with Euclidean distance and Ward's method). Heatmaps were used to highlight the associations between the clustering and the expression levels of the genes. The shrinkage approach [6] was used to assess differences in gene expression levels between two groups of interest, using local false discovery rate as method to control false positives. When this approach had been believed inaccurate [7], [8] we used a permutation approach on filtered probe sets (filtering out probe sets with small variance across samples; 90% of the probe sets removed) with tests based on standardized rank sum Wilcoxon statistics and we control false positives with the method of Benjamini and Hochberg [9]. Results from these two approaches were considered significant if they reached local false discovery rates $<0.05$ or adjusted p-values $<0.05$. For each probe fold changes were calculated as either differences (mRNA) or ratio (ncRNA) of the mean intensity in the compared groups.

Analyses of microRNAs expression were performed on 847 human microRNA probe sets, analyses of small nucleolar RNAs (snoRNAs) expression were performed on 922 human snoRNAs probe sets.

Gene expression profiles of all 143 samples in the study cohort were used to build a classifier. We

used LASSO [10] as prediction method. The method is implemented in the package CMA [11] which is available through bioconductor (www.bioconductor.org). Prediction accuracy was assessed using 5 fold cross validation (10 iterations) with stratified sampling. Hyperparameter tuning was carried out using an inner loop of 3 fold cross validation. Enrichment of relevant signatures previously published was analyzed using Gene Set Enrichment Analysis (GSEA) software [12, 13].

## Quantitative assay of ncRNA

MiRNAs expression data were validated for 4 miRNAs of interest measuring the amount of mature miRNA by TaqMan® MicroRNA assays (Applied Biosystems, Foster City, CA, USA). MiRNAs tested were hsa-miR-125b (Assay ID 000449), hsa-miR-125b-2* (Assay ID 002158), hsa-miR-99a (Assay ID 000435), hsa-miR-let-7c (Assay ID 000379) and RNU6B was assessed as endogenous control (Assay ID 001093). cDNA was generated by TaqMan® MicroRNA Reverse Transcription (RT) Kit (Applied Biosystems, Foster City, CA, USA) starting from 5 ng of total RNA. PCR was carried out with the 7900HT Fast Real Time PCR system (Applied Biosystems, USA). Relative expression was calculated using the comparative Ct method [14] and using an ERG-related patient as reference.

SnoRNAs expression of 5 representative snoRNAs in the PWS region (SNORD116-11, -15, -22, -24 and SNORD109A) was validated by miScrpt PCR System (Qiagen). RNU6B was used as endogenous control. cDNA was generated by miScript II RT kit (HiFlex buffer) starting from 10ng of total RNA and following manufacturer's instructions. qRT-PCR was carried out using the miScript Universal primer and the following ncRNAs specific primers: RNU6B 5'-G CAAGGATGACACGCAAATT-3'; SNORD116-11 5'-TG ATGACTTCCATACGTGGG-3'; SNORD116-15 5'-CGT CATCCTCGTCAAA-3'; SNORD116-22 5'- CCATATG TACATTCCTTGG-3'; SNORD116-24 5'- CTATACCGT CATCTTCGTTGAACTGAG-3'; SNORD109A 5'- GA TGATGAGAATAATTGTCTGAGGATG-3'. Relative expression was assessed by comparative Ct method using an ERG-related patient as reference.

## Quantitative assay of gene ERG

cDNA was synthesised starting from 1μg of total RNA by RevertAid H Minus Reverse Transcriptase (Thermo Scientific) in the presence of RiboLock RNase inhibitor (Thermo Scientific) and using oligo(dT). Gene expression analysis was performed by TaqMan gene expression assays in StepOnePlus Real-Time PCR Systems (Applied Biosystems). Hs 01554629-m1 assay was used to assess *ERG* gene expression (target sequence between exon 3 and exon 4 of *ERG* transcript variant 1, NM_182918.3). *OAZ1* was used as reference gene (Hs 00427923_m1). For each sample, assays were run in duplicate using 2.5ng of cDNA. Relative gene expression quantitation was assessed by comparative Ct method using an ERG-related patient with *ERG* deletion as reference.

## Characterization of ERG intragenic deletions

Breakpoints on genomic DNA were investigated in patients' samples by long-range PCR using PCR Extender System (5 Prime). PCR condition was setup to allow up to 30kb template amplification starting from 500ng of gDNA. Forward primers mapping in the genomic coding sequence of exon10 (5'-GTAGTAAGTGCCCAGATGA GAAGG-3') and reverse primers mapping in the coding sequence of exon2 (5'-TATCAGTTGTGAGTGAGGACC AGT-3') were used. To better characterize the breakpoints in patients' samples a second PCR was run on 100ng gDNA using forward primer (5'-CCTTGCTTTCTATTC TCACAGTCC-3') and reverse primer (5'-TAGAACTTG TGGGCTGTTACCTTT-3').

Expression of deleted *ERG* transcripts in patients samples were investigated by PCR on cDNA (10ng) using forward primer in exon2 (5'-ACACCTGGCTAAGACAG AGATGAC-3') and reverse primer in exon10 (5'-TTATC GTAGTTCATGTTGGGTTTG-3').

PCR products were analyzed by Sanger sequencing (exons number based on *ERG* transcript variant 1, NM_182918.3).

## Multiplex ligation-dependent Probe Amplification (MLPA) analysis

MLPA analysis has been developed according to the manufacturer's protocol using SALSA MLPA probemix P335-B1 ALL-IKZF1 kit (MRC-Holland). Briefly, for each MLPA reaction 50-250ng of DNA sample was used. PCR amplification fragments ranging between 64 and 500nt in length were analyzed by capillary electrophoresis on ABI-3130 Genetic Analyzer (Applied Biosystems). For data analysis, Coffalyser.NET version v120309.150 was used.

## Western blot analysis

Whole cells lysates of leukemia samples at diagnosis were obtained from cryopreserved bone marrow mononuclear cells using RIPA lysis buffer. Proteins were resolved in 4-15% Criterion™ TGX™ Precast Gels (Bio-Rad) and transferred to nitrocellulose membrane. Membrane was blocked with 5% nonfat milk in PBS and 0.1% Tween-20 and labeled with primary anti-ERG rabbit monoclonal antibody [EPR3864(2)] (ab133264, Abcam; used at 1:2,000 dilution) followed by incubation

with secondary HRP-conjugated antibody (NA934, GE Healthcare Life Sciences). Peroxidase activity was detected with Clarity™ Western ECL Blotting Substrate (#170-5061, Bio-Rad).

Membrane was stripped of antibodies with ReBlot Plus Mild Antibody Stripping Solution (2502, Millipore), blocked and re-probed with anti-β-actin-HRP (A3854, Sigma; used at 1:20,000 dilution) as loading control. Peroxidase activity was detected with Amersham™ ECL™ Western Blotting Detection Reagents (RPN2109, GE Healthcare Life Sciences).

### Statistical analysis

Event Free Survival (EFS) and overall survival were estimated according to Kaplan-Meier, with Greenwood standard error and with the log-rank test for comparison; Cumulative Relapse Incidence (CRI) was estimated adjusting for competing risks of other events and compared with the Grey test. The starting point of the observation time was the date of diagnosis; events considered were: relapse for CRI; relapse, resistance, death or second neoplasm, whichever occurred first, for EFS; death for any cause for overall survival. To assess associations between patients' features, the Chi-Square test was applied. GraphPad Prism software and SAS 9.2 were used for analyses (GraphPad Software, La Jolla, CA, USA).

## REFERENCES


1. Conter V, Bartram CR, Valsecchi MG, Schrauder A, Panzer-Grümayer R, Möricke A, Aricò M, Zimmermann M, Mann G, De Rossi G, Stanulla M, Locatelli F, Basso G, et al. Molecular response to treatment redefines all prognostic factors in children and adolescents with B-cell precursor acute lymphoblastic leukemia: results in 3184 patients of the AIEOP-BFM ALL 2000 study. Blood. 2010; 115:3206–14.
2. Campo Dell'Orto M, Zangrando A, Trentin L, Li R, Liu WM, te Kronnie G, Basso G, Kohlmann A. New data on robustness of gene expression signatures in leukemia: comparison of three distinct total RNA preparation procedures. BMC Genomics. 2007; 8:188.
3. Edgar R, Domrachev M, Lash AE. Gene Expression Omnibus: NCBI gene expression and hybridization array data repository. Nucleic Acids Res. 2002; 30:207–10.
4. Irizarry RA, Hobbs B, Collin F, Beazer-Barclay YD, Antonellis KJ, Scherf U, Speed TP. Exploration, normalization, and summaries of high density oligonucleotide array probe level data. Biostatistics. 2003; 4:249–64.
5. Johnson WE, Li C, Rabinovic A. Adjusting batch effects in microarray expression data using empirical Bayes methods. Biostatistics. 2007; 8:118–27.
6. Opgen-Rhein R and Strimmer K. Accurate ranking of differentially expressed genes by a distribution-free shrinkage approach. Stat Appl Genet Mol Biol. 2007; 6:Article9. doi: 10.2202/1544-6115.1252.
7. Efron B. Large-Scale Simultaneous Hypothesis Testing. The Choice of a Null Hypothesis J Amer Statist Assoc. 2004; 99:96–104.
8. Strimmer K. A unified approach to false discovery rate estimation. BMC Bioinformatics. 2008; 9:303.
9. Benjamini Y, Hochberg Y. Controlling the False Discovery Rate: A Practical and Powerful Approach to Multiple Testing. J R Stat Soc B. 1995; 57:289–300. Statistical Methodology.
10. Tibshirani R. Regression Shrinkage and Selection via the Lasso. J R Stat Soc B. 1996; 58:267–88. Statistical Methodology.
11. Slawski M, Boulesteix AL and Bernau C. CMA: Synthesis of microarray-based classification. R package version 1.6.0. 2009.
12. Subramanian A, Tamayo P, Mootha VK, Mukherjee S, Ebert BL, Gillette MA, Paulovich A, Pomeroy SL, Golub TR, Lander ES, Mesirov JP. Gene set enrichment analysis: a knowledge-based approach for interpreting genome-wide expression profiles. Proc Natl Acad Sci USA. 2005; 102:15545–50.
13. Mootha VK, Lindgren CM, Eriksson KF, Subramanian A, Sihag S, Lehar J, Puigserver P, Carlsson E, Ridderstråle M, Laurila E, Houstis N, Daly MJ, Patterson N, et al. PGC-1alpha-responsive genes involved in oxidative phosphorylation are coordinately downregulated in human diabetes. Nat Genet. 2003; 34:267–73.
14. Livak KJ and Schmittgen TD. Analysis of relative gene expression data using real-time quantitative PCR and the 2(-Delta Delta C(T)) Method. Methods. 2001; 25:402-08.
15. Harvey RC, Mullighan CG, Wang X, Dobbin KK, Davidson GS, Bedrick EJ, Chen IM, Atlas SR, Kang H, Ar K, Wilson CS, Wharton W, Murphy M, et al. Identification of novel cluster groups in pediatric high-risk B-precursor acute lymphoblastic leukemia with gene expression profiling: correlation with genome-wide DNA copy number alterations, clinical characteristics, and outcome. Blood. 2010; 116:4874–84.
16. Castle JC, Armour CD, Löwer M, Haynor D, Biery M, Bouzek H, Chen R, Jackson S, Johnson JM, Rohl CA, Raymond CK. Digital genome-wide ncRNA expression, including SnoRNAs, across 11 human tissues using polyA-neutral amplification. PLoS One. 2010; 5:e11779.

17. Zhang J, McCastlain K, Yoshihara H, Xu B, Chang Y, Churchman ML, Wu G, Li Y, Wei L, Iacobucci I, Liu Y, Qu C, Wen J, et al, and St. Jude Children's Research Hospital–Washington University Pediatric Cancer Genome Project. Deregulation of DUX4 and ERG in acute lymphoblastic leukemia. Nat Genet. 2016; 48:1481–89.

18. Tursky ML, Beck D, Thoms JA, Huang Y, Kumari A, Unnikrishnan A, Knezevic K, Evans K, Richards LA, Lee E, Morris J, Goldberg L, Izraeli S, et al. Overexpression of ERG in cord blood progenitors promotes expansion and recapitulates molecular signatures of high ERG leukemias. Leukemia. 2015; 29:819–27.

## SUPPLEMENTARY SCHEME, FIGURES AND TABLES

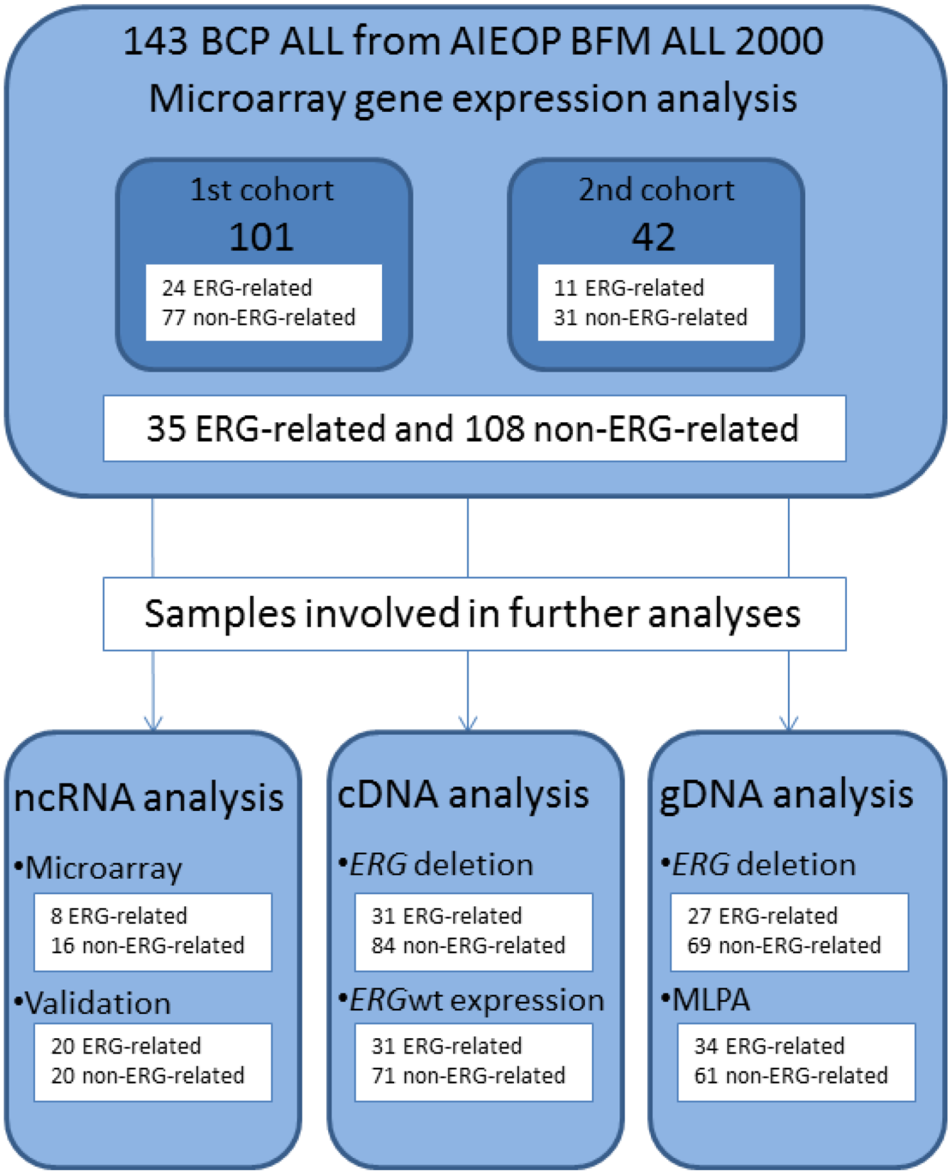


**Scheme 1: Scheme indicating the samples that were used in the various analysis.**

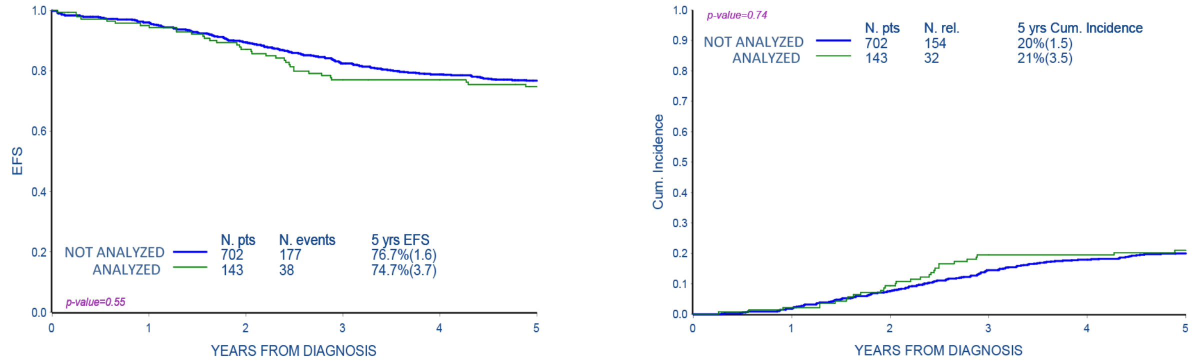


**Supplementary Figure 1: Event free survival (EFS) and cumulative incidence of relapse of B-others patients enrolled in the AIEOP ALL 2000 study protocol included (143 analyzed) and not included (702 not analyzed) in the present study (diagnosis time-window of included patients was considered).**

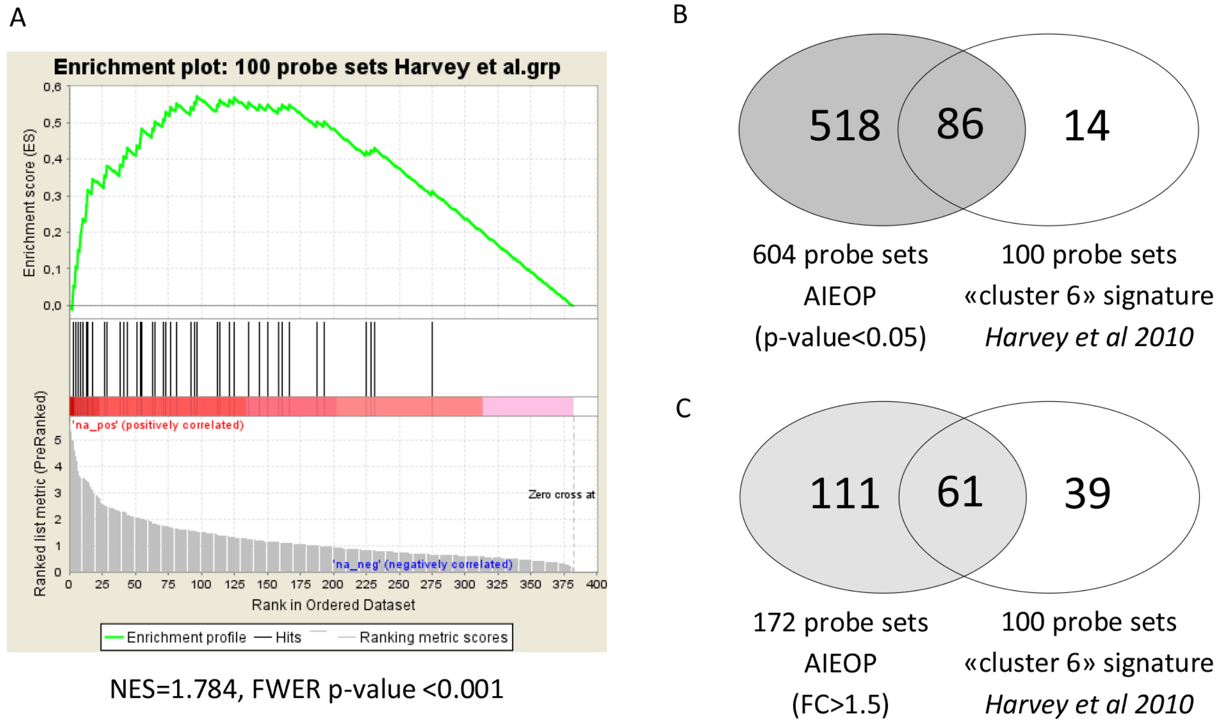


**Supplementary Figure 2: High similarity between the signature identified in the AIEOP B-others cohort analyzed in this study and the ERG-related signature published by Harvey et al. [15]. (A)** Up-regulated probe sets (604) in ERG-related patients identified by class comparison analysis in the AIEOP cohort were analyzed for enrichment of the ERG-related signature (100 probe sets) published by Harvey et al. as specifically upregulated in "cluster 6" in the high risk St. Jude cohort (Children's Oncology Group, COG). When uploaded for analysis, the 604 probe sets list was collapsed to 382 gene symbols while the 100 probe sets list was collapsed to 61 gene symbols by the GSEA software. Forty-one genes were found in common between the two lists and 25 genes were in the enrichment core. NES=1.784; FWER p-value<0.001. **(B)** Overlap between the 100 probe sets of the "cluster 6" signature and 604 up-regulated probe sets in the AIEOP ERG-related cohort. **(C)** Overlap between the 100 probe sets of the "cluster 6" signature and 172 most up-regulated probe sets (FC>1.5) in the AIEOP ERG-related cohort.

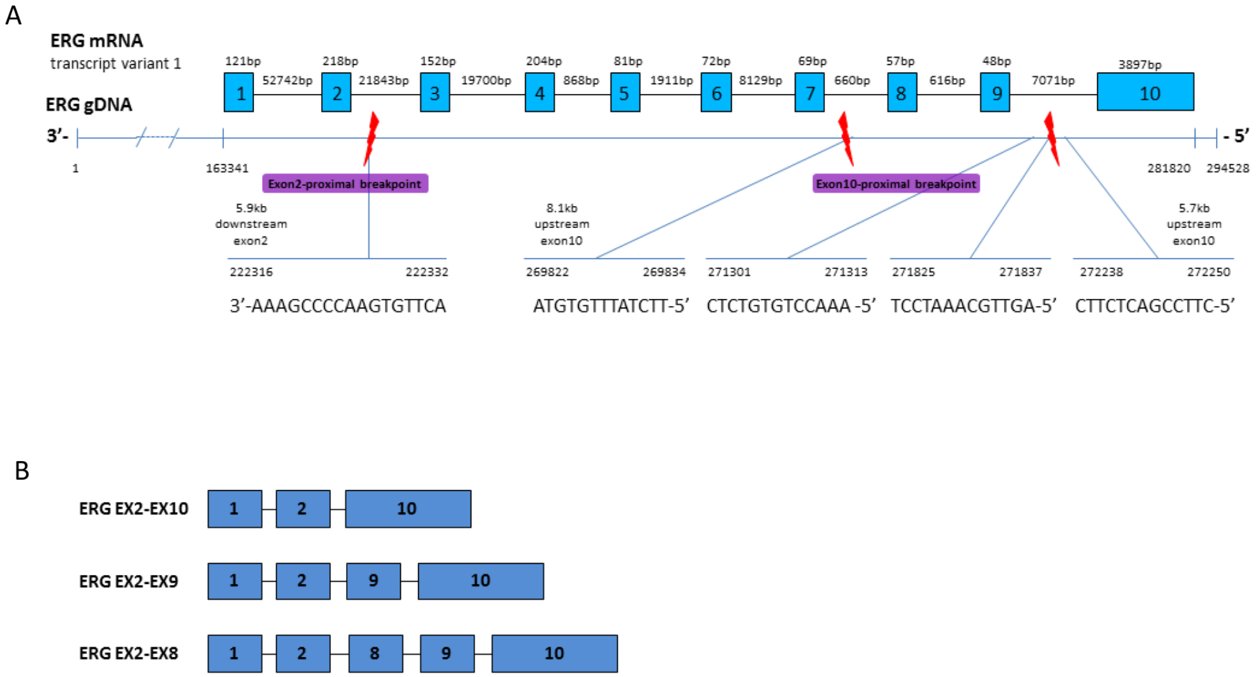


**Supplementary Figure 3: Scheme of *ERG* intragenic breakpoints and *ERG* deleted transcripts. (A)** Schematic representation of exons and introns in the *ERG* (gene ID 2078; NG_029732.1) genomic sequence according to *ERG* isoform 1 (NM_182918). Position of exon2-proximal breakpoints and the most frequent exon10-proximal breakpoints identified in the *ERG* deleted patients are shown (see also Supplementary Table 5). **(B)** Schematic representation of *ERG* deleted transcripts found in *ERG* deleted patients according to *ERG* isoform 1 (NM_182918).

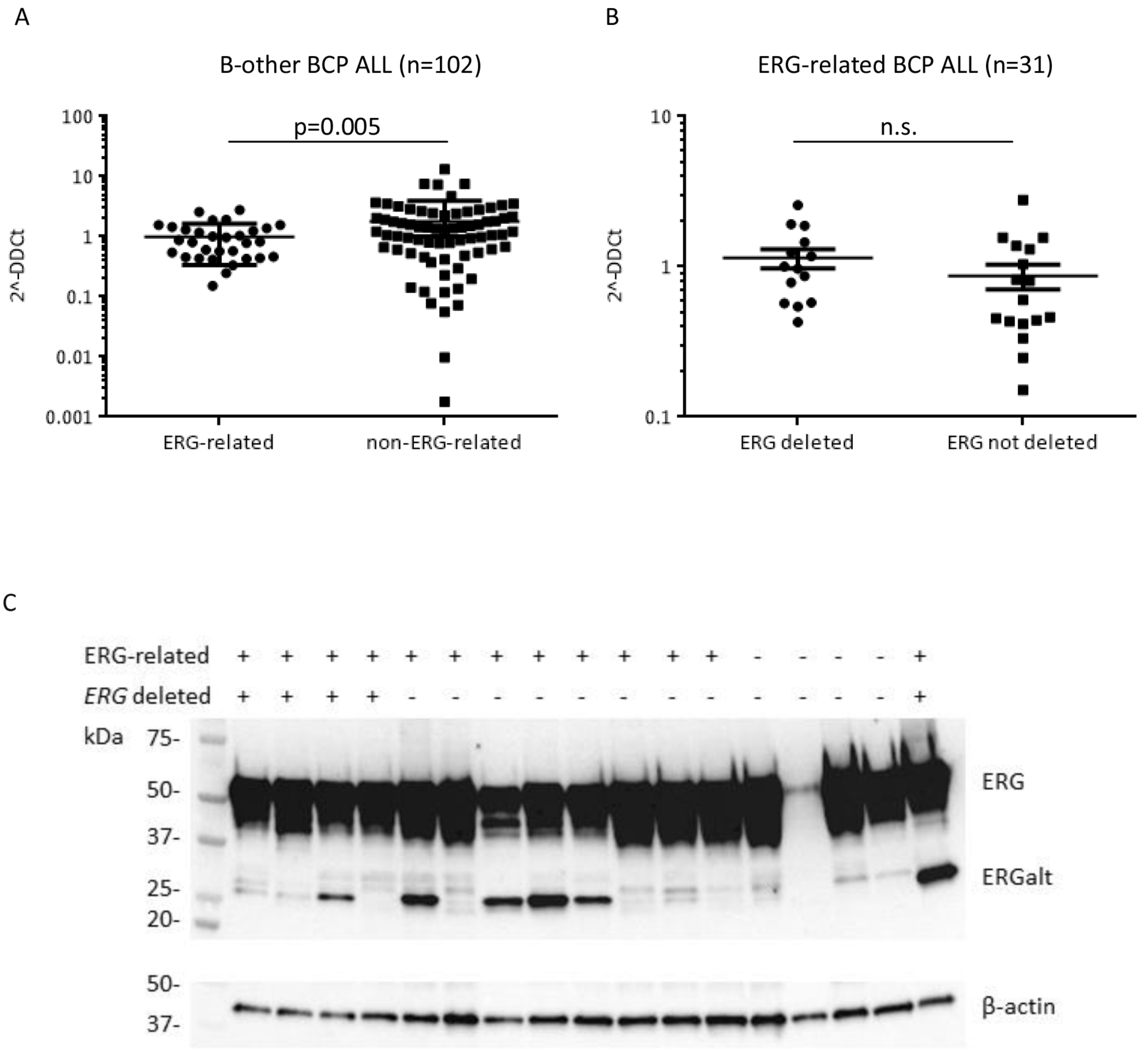


**Supplementary Figure 4: Analysis of *ERG* wild type expression by qRT-PCR in B-others cohort.** **(A)** Expression of *ERG* wt in the B-others cohort according to the ERG-related signature. Expression was significantly higher in non-ERG-related patients when compared to ERG-related (Mean with SEM is shown, unpaired t-test with Welch's correction, p-value=0.005). **(B)** Expression of *ERG* wt in the ERG-related patients according to the presence of *ERG* intragenic deletions. No significative difference was found among patients (Mean with SEM is shown). **(C)** Western blot analysis of ERG protein expression in a representative group of ERG-related patients samples with (5) and without (8) *ERG* intragenic deletion, and in a small group of non-ERG-related samples (4). An additional short ERG isoform, supposedly the ERGalt protein described by Zhang and colleagues [17], was detected in 6 out of 13 ERG-related patients.

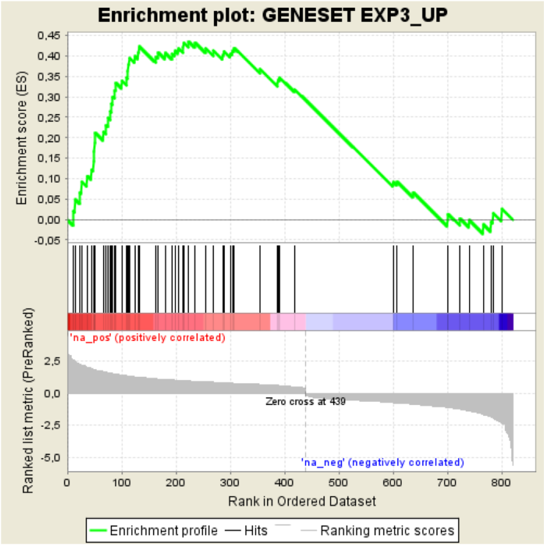


NES=2.122, FWER p-value <0.001

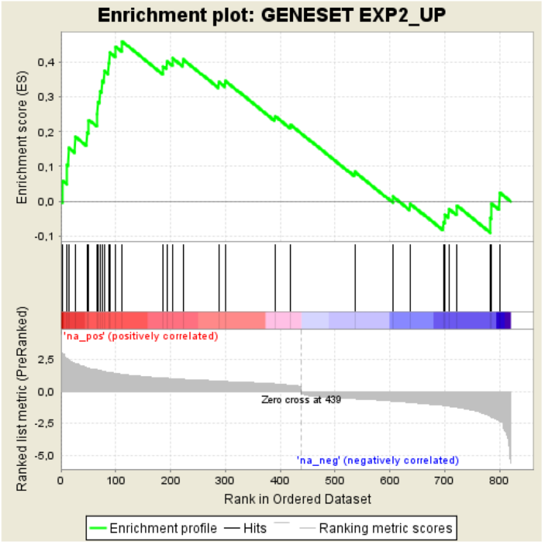


NES=1.944, FWER p-value =0.006

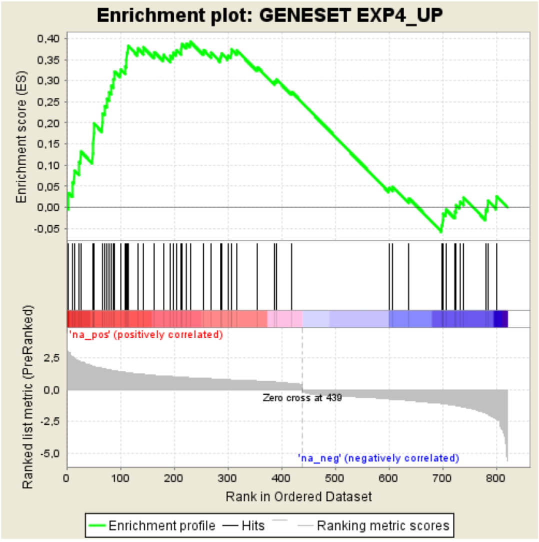


NES=1.917, FWER p-value =0.009

**Supplementary Figure 5: Genes up-regulated in non-ERG-related patients are enriched in ERG downstream target genes.** The gene list of 1323 probe sets differently expressed between ERG-related and non-ERG-related patients was analyzed by GSEA for enrichment of ERG up-regulated downstream targets recently published [18]. A gene sets matrix was build including 4 gene sets of genes up-regulated in human cord blood CD34+ cells overexpressing ERG (EXP-UP) and 4 genesets of genes down-regulated during the same experiments (EXP-DOWN). Analysis was run on the 1323 gene list pre-raked according to fold change. Three out of 4 genesets (EXP2-UP, EXP3-UP, EXP4-UP) of ERG up-regulated genes were found to be significantly enriched among the genes highly expressed in non-ERG-related patients.

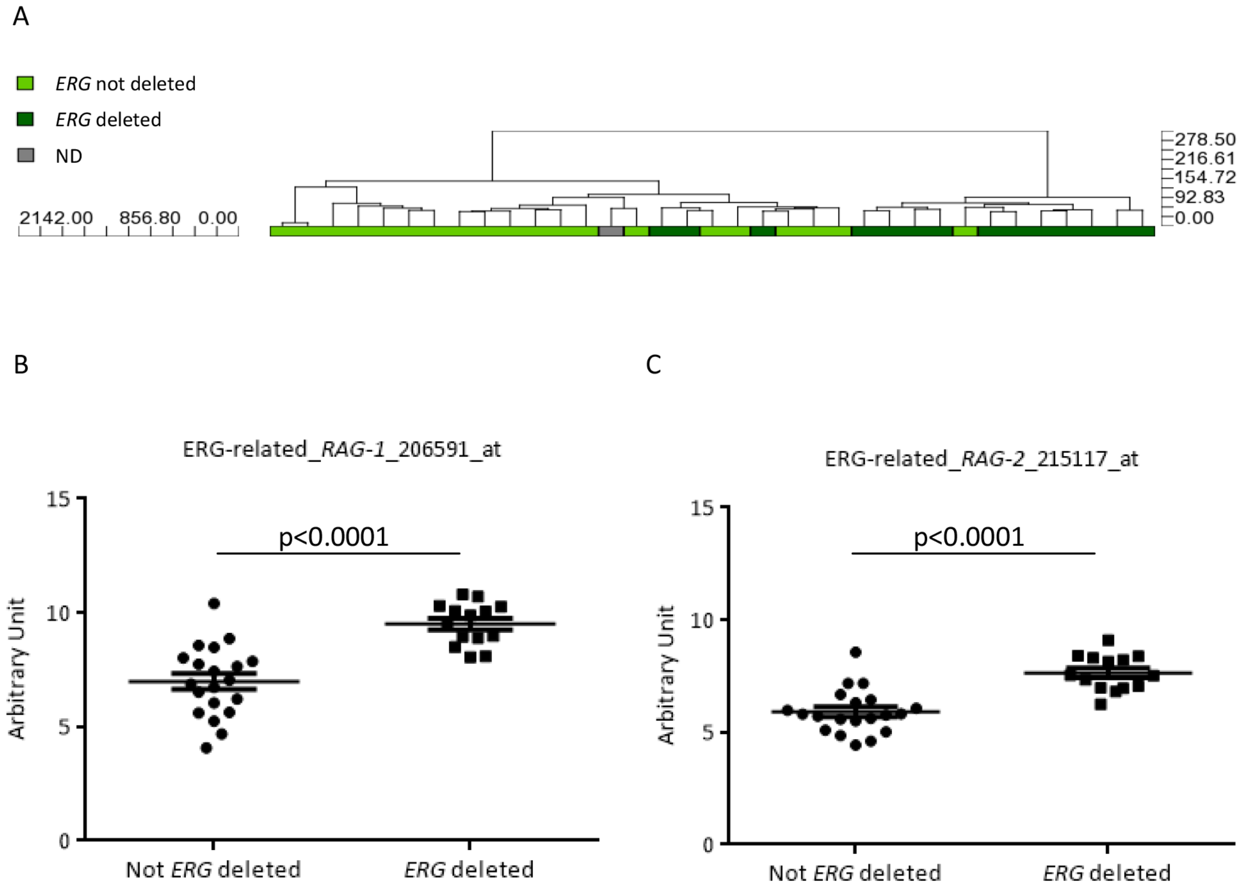


**Supplementary Figure 6: Distinct gene expression signature among ERG-related patients with and without *ERG* intragenic deletions.** **(A)** Dendrogram represents an unsupervised hierarchical clustering of 35 ERG-related patients according to gene expression profile (Affymetrix U133 Plus 2.0 array). Patients carrying the *ERG* intragenic deletion cluster apart from the ERG-related patients without the deletion. **(B-C)** Expression level of Recombination activating genes: *RAG-1* **(A)** and *RAG-2* **(B)** gene expression, measured by microarray (probe sets 206591_at and 215117_at respectively), was significantly higher in ERG-related patients with *ERG* intragenic deletion when compared to those without the deletion (Mean with SEM is shown, unpaired t-test with Welch's correction, p-value<0.0001).

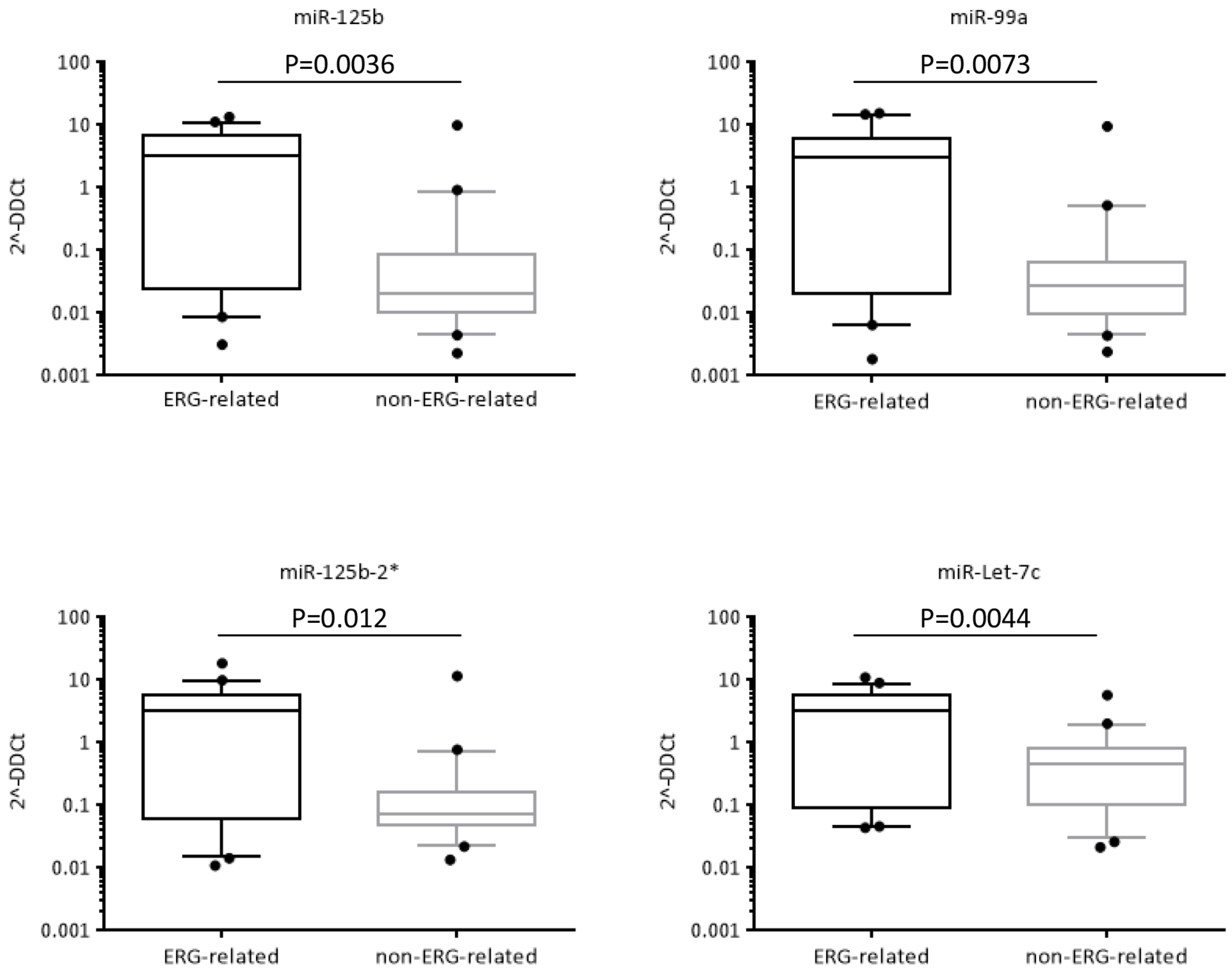


**Supplementary Figure 7: qRT-PCR validation of differentially regulated microRNAs. (A)** Validation by qRT-PCR analysis of mature miRNA expression in 40 samples (20 ERG-related and 20 non-ERG-related) included in the 143 samples study group and not included in the miRNA microarrays analysis. MiRNAs in the miR-125b-2 cluster (miR-125b, miR-125b-2*, miR-99a and miR-let-7c; hosted in LIN000478 host gene) confirmed to be highly expressed in the most of ERG-related patients (Box and whisker plots showing the median value and 10-90 percentiles, unpaired t-test with Welch's correction).

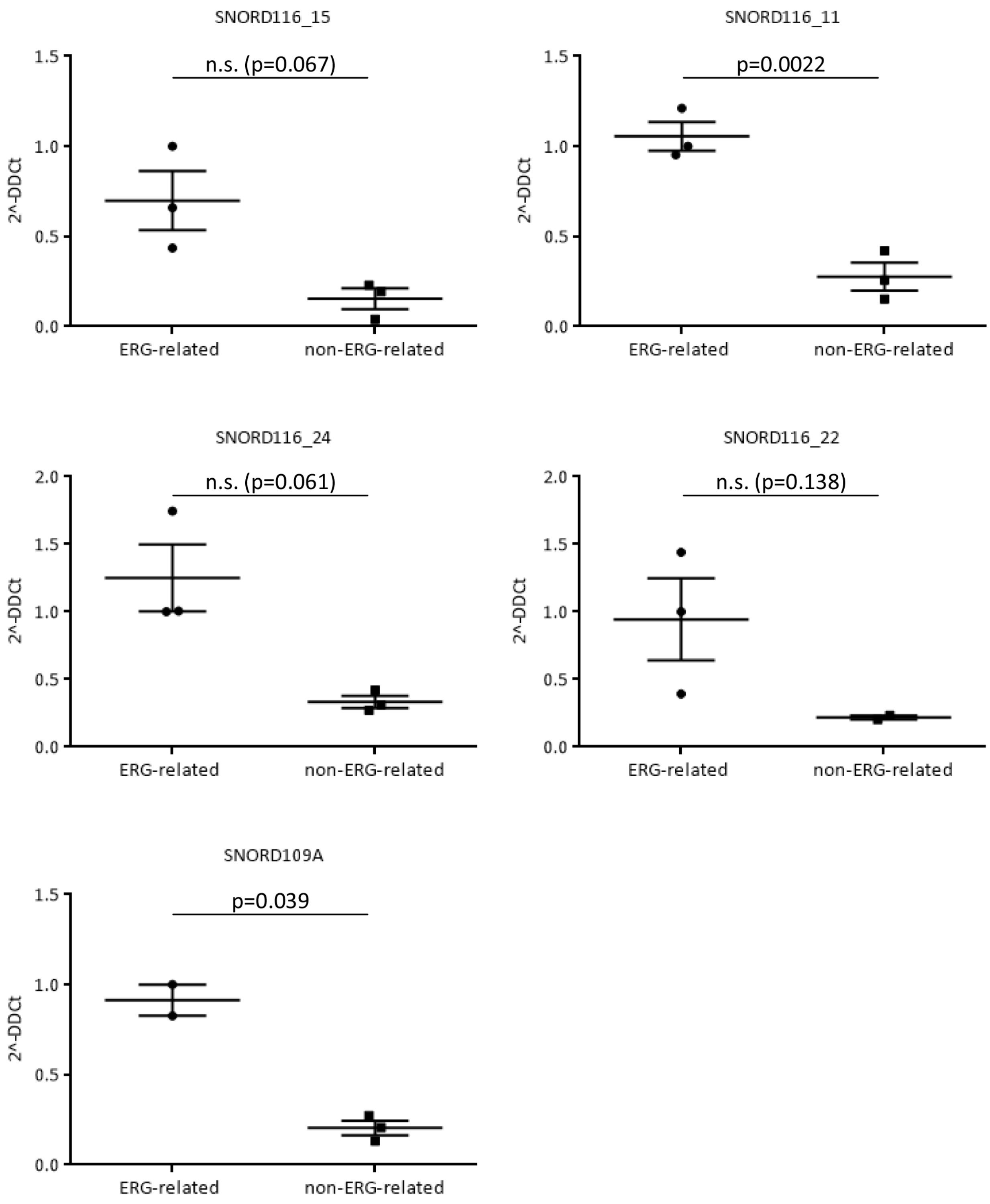


**Supplementary Figure 8: qRT-PCR validation of differentially regulated snoRNAs.** Validation by qRT-PCR analysis of snoRNAs expression in 6 samples (3 ERG-related and 3 non-ERG-related) out of 24 profiled with miRNA microarrays. SnoRNAs confirmed to be highly expressed in ERG-related patients (Mean with SEM is shown, unpaired t-test with Welch's correction).

**Supplementary Table 1: Clinical features of B-others patients enrolled in the AIEOP ALL 2000 study protocol included (143 analyzed) and not included (702 not analyzed) in the present study (diagnosis time-window of included patients was considered)**

| | ANALYZED (%) | NOT ANALYZED (%) |
|---|---|---|
| **Total n. of patients** | 143 | 702 |
| **GENDER** | | |
| Male | 54.6 | 54.3 |
| Female | 45.4 | 45.7 |
| *p-value=0.95* | | |
| **AGE** | | |
| 1-5 yrs | 51.0 | 56.4 |
| 6-9 yrs | 25.9 | 20.2 |
| 10-17 yrs | 23.1 | 23.4 |
| *p-value=0.30* | | |
| **WBC** | | |
| <20000 | 52.4 | 74.2 |
| 20-100000 | 33.6 | 21.1 |
| ≥100000 | 12.6 | 4.4 |
| Not known | 1.4 | 0.3 |
| *p-value(not known excluded) <0.001* | | |
| **PREDNISONE RESPONSE** | | |
| Good | 82.5 | 92.0 |
| Poor | 16.1 | 7.1 |
| Not known | 1.4 | 0.9 |
| *p-value (not known excluded) <0.001* | | |
| **MRD STRATIFICATION** | | |
| Standard | 25.2 | 24.5 |
| Medium | 47.6 | 46.9 |
| High | 11.9 | 5.7 |
| Not known | 15.4 | 22.9 |
| *p-value (not known excluded)=0.06* | | |
| **FINAL RISK** | | |
| Standard | 23.8 | 23.1 |
| Medium | 53.1 | 64.8 |
| High | 23.1 | 12.1 |
| *p-value=0.002* | | |

WBC, white blood cells; yrs, years; MRD, minimal residual diseases.

**Supplementary Table 2: Top differentially regulated probe sets identified by class comparison analysis between ERG-related and non-ERG-related groups.**

**See Supplementary File 1**

**Supplementary Table 3: Summary of the events**

| | Non-ERG-related | | ERG-related | | ERG-related without *ERG* deletion | | ERG-related with *ERG* deletion | |
|---|---|---|---|---|---|---|---|---|
| | **N** | **%** | **N** | **%** | **N** | **%** | **N** | **%** |
| **Total n. of patients** | 108 | | 35 | | 20 | | 14 | |
| **Resistant** | 0 | | 0 | | 0 | | 0 | |
| **Death IND** | 2 | 1.9 | 0 | | 0 | | 0 | |
| **Relapses** | 29 | 26.8 | 3 | 8.6 | 3 | 15.0 | 0 | |
| **Death in CCR** | 3 | 2.8 | 0 | | | | | |
| After chemo | 2 | 1.9 | | | | | | |
| After HSCT | 1 | 0.9 | | | | | | |
| **SMN** | 0 | | 1 | 2.9 | 1 | 5.0 | 0 | |
| **Alive in CCR** | 74 | 68.5 | 31 | 88.6 | 16 | 80.0 | 14 | 100 |

To the left, events recorded in the 143 B-other patients according to the distinction in the unsupervised gene expression analyses: ERG-related, non-ERG-related. To the right, events in the 34 ERG-related patients according to the presence of *ERG* intragenic deletion (one ERG-related patient not analyzed for *ERG* intragenic deletion was excluded). IND, Induction; CCR, Continuous Complete Remission; HSCT, Hematopoietic Stem Cell Transplantation; SMN, Secondary Malignant Neoplasm.

**Supplementary Table 4: Probe sets classifying ERG-related patients.**

**See Supplementary File 2**

**Supplementary Table 5: Summary of aberrations on *ERG* gDNA and mRNA identified in ERG-related patients carrying the *ERG* intragenic deletion.**

**See Supplementary File 3**

**Supplementary Table 6: Most differentially regulated probe sets identified by class comparison analysis between ERG-related patients with and without *ERG* intragenic deletion.**

**See Supplementary File 4**

**Supplementary Table 7: List of aberrations identified in the 95 patients analyzed by SALSA MLPA P335-B1 ALL-IKZF1.**

**See Supplementary File 5**

**Supplementary Table 8: Complete sequence and structure of snoRNAs in the SNORD116 cluster are listed.**

**See Supplementary File 6**
