# Supplementary material for: High expression of miR-125b-2 and SNORD116 noncoding RNA clusters characterize ERG-related B cell precursor acute lymphoblastic leukemia
Source: Oncotarget. 2017 Mar 21;8(26):42398–413. doi: 10.18632/oncotarget.16392 (PMC5522075; doi:10.18632/oncotarget.16392)
Supplement: Supplementary file 3 [file oncotarget-08-42398-s003.pdf]

Supplementary table 4: Probe sets classifying ERG-related patients

Probe set	Gene symbol		Probe set	Gene symbol
228863_at	PCDH17		219789_at	NPR3
227289_at	PCDH17		239826_at	NA
205656_at	PCDH17		237261_at	ANGPT2
1554343_a_at	STAP1		235751_s_at	VMO1
209602_s_at	GATA3		219225_at	PGBD5
230537_at	NA		207345_at	FST
240758_at	NA		205423_at	AP1B1
219489_s_at	NXN		227358_at	ZBTB46
203921_at	CHST2		1563113_at	UBR4
239956_at	NA		227370_at	FAM171B
206756_at	CHST7		213272_s_at	TMEM159
235146_at	TMCC3		38918_at	SOX13
202409_at	IGF2 /// INS-IGF2		236501_at	SALL4
244665_at	NA		210258_at	RGS13
206067_s_at	WT1		226499_at	NRARP
202747_s_at	ITM2A		209736_at	SOX13
209603_at	GATA3		244509_at	GPR155
233038_at	NA		203448_s_at	TERF1
215146_s_at	TTC28		229677_at	SLC39A3
234196_at	NA		217606_at	NA
233225_at	NA		211209_x_at	SH2D1A
235343_at	VASH2		200753_x_at	SFRS2
225611_at	MAST4		213060_s_at	CHI3L2
231600_at	CLEC12B		219790_s_at	NPR3
209959_at	NR4A3		238091_at	LOC100506388
232227_at	LOC100505976		1554258_a_at	DNAJC5B
235968_at	AGAP1		207815_at	PF4V1
213058_at	TTC28		207518_at	DGKE
229091_s_at	CCNJ		35156_at	R3HCC1
205572_at	ANGPT2		203110_at	PTK2B
226489_at	TMCC3		201700_at	CCND3
238865_at	PABPC4L		214716_at	BMP2K
209875_s_at	SPP1		37170_at	BMP2K
219470_x_at	CCNJ		221558_s_at	LEF1
1556592_at	NA		201889_at	FAM3C
225369_at	ESAM		210948_s_at	LEF1
223627_at	MEX3B		230069_at	SFXN1
225532_at	CABLES1		231793_s_at	CAMK2D
229661_at	SALL4		209781_s_at	KHDRBS3
216979_at	NR4A3		225019_at	CAMK2D
231357_at	CLEC12B		220952_s_at	PLEKHA5
201200_at	CREG1		203066_at	CHST15
219227_at	CCNJL		227486_at	NT5E
242218_at	PPARD		205289_at	BMP2
1555216_a_at	LOC645722		205290_s_at	BMP2
224374_s_at	EMILIN2			
