# Supplementary material for: High expression of miR-125b-2 and SNORD116 noncoding RNA clusters characterize ERG-related B cell precursor acute lymphoblastic leukemia
Source: Oncotarget. 2017 Mar 21;8(26):42398–413. doi: 10.18632/oncotarget.16392 (PMC5522075; doi:10.18632/oncotarget.16392)
Supplement: Supplementary file 4 [file oncotarget-08-42398-s004.pdf]

Supplementary table 5: Summary of aberrations on *ERG* gDNA and mRNA identified in ERG-related patients carrying the *ERG* intragenic deletion.

SAMPLE	N. BREAK POINTS	EXON 2-PROXIMAL BREAKPOINT		INSERT	EXON 10-PROXIMAL BREAKPOINT			ABERRANT ERG TRANSCRIPTS
		POSITION	SEQUENCE	SEQUENCE	SEQUENCE	POSITION	INTRON	
1	1	222327	AAAGCCCCAAGT	GGCCC	CCTAAACGTTGAGGCCAA	271826	9	EX2-EX10
2	1	222327	AAAGCCCCAAGT	AGAGGGCC	AACGTTGAGGCCAA	271830	9	EX2-EX10
3	1	222331	AAAGCCCCAAGTGTTC	CCCCCCAG	CGTTGAGGCCAA	271832	9	EX2-EX10
4	1**	ND	ND	ND	ND	ND	ND	EX2-EX10
5	1**	ND	ND	ND	ND	ND	ND	EX2-EX10, EX2-EX9, EX2-EX8
6	1	222322	AAAGCCC	ATC	TGTTTATCTT	269825	7	EX2-EX10*, EX2-EX9, EX2-EX8
7	1	222329	AAAGCCCCAAGTGT	CCCCCCCT	TGTGTGTCAGTCTCTGTGTCCAAA	271290	9	EX2-EX10
8	2**	ND	ND	ND	ND	ND	ND	EX2-EX9*, EX2-EX8*
		ND	ND	ND	ND	ND	ND	
9	4	222332	AAAGCCCCAAGTGTTCA	AACC	CTTCTCAGCCTTC	272238	9	EX2-EX10*, EX2-EX9, EX2-EX8
		222332	AAAGCCCCAAGTGTTCA	no insert	ATTTCCTAAACGTTGAGGCCAA	271822	9	
		222325	AAAGCCCCAA	no insert	ACGTTGAGGCCAA	271831	9	
		222327	AAAGCCCCAAGT	CCCCCCCCGGG	TCTTCTCAGCCTTC	272237	9	
10	6	222324	AAAGCCCCA	GGTAGG	CTCTGTGTCCAAA	271301	9	EX2-EX10, EX2-EX9, EX2-EX8
		222329	AAAGCCCCAAGTGT	CCCC	GTCTCTTCTCAGCCTTC	272234	9	
		222321	AAAGCC	TCGTTCGGCG	TCTTCTCAGCCTTC	272237	9	
		222330	AAAGCCCCAAGTGTT	TAGGGAG	ACGTTGAGGCCAA	271831	9	
		222326	AAAGCCCCAAG	CGTTA	TATTTCCTAAACGTTGAGGCCAA	271821	9	
		222331	AAAGCCCCAAGTGTTC	no insert	CCTAAACGTTGAGGCCAA	271826	9	
11	14	222323	AAAGCCCC	CGGCG	CTTCTCAGCCTTC	272238	9	EX2-EX10, EX2-EX9, EX2-EX8
		222327	AAAGCCCCAAGT	CGAA	CTTCTCAGCCTTC	272238	9	
		222327	AAAGCCCCAAGT	C	CTTCTCAGCCTTC	272238	9	
		222329	AAAGCCCCAAGTGT	CCCTCCCC	GTCTCTTCTCAGCCTTC	272234	9	
		222323	AAAGCCCC	C	GTCTCTTCTCAGCCTTC	272234	9	
		222323	AAAGCCCC	no insert	CGTCTCTTCTCAGCCTTC	272233	9	
		222327	AAAGCCCCAAGT	no insert	GTCTCTTCTCAGCCTTC	272234	9	
		222327	AAAGCCCCAAGT	TCCC	GTCTCTTCTCAGCCTTC	272234	9	
		222328	AAAGCCCCAAGTG	CCTCCTAG	GGCCAA	271838	9	
		222329	AAAGCCCCAAGTGT	CGAGG	CTTCTCAGCCTTC	272238	9	
		222332	AAAGCCCCAAGTGTTCA	GGC	GTCTCTTCTCAGCCTTC	272234	9	
		222332	AAAGCCCCAAGTGTTCA	GGG	CTCTTCTCAGCCTTC	272236	9	
		222331	AAAGCCCCAAGTGTTC	CCCGAGG	CCTAAACGTTGAGGCCAA	271826	9	
		222327	AAAGCCCCAAGT	TCACACCC	CAGTCTCTGTGTCCAAA	271297	9	
12	ND	ND	ND	ND	ND	ND	ND	EX2-EX10
13	ND	ND	ND	ND	ND	ND	ND	EX2-EX10
14	ND	ND	ND	ND	ND	ND	ND	EX2-EX10

Different breakpoints, position on the gene *ERG*, sequences and inserts identified by analysis of gDNA in 11 patients are listed. Type of aberrant *ERG* transcripts identified by analysis of mRNA in 14 patients are listed. *ERG* gene ID 2078, NG_029732.1; exons numbering according to *ERG* isoform 1 (NM_182918). **ERG* transcripts inferred by the size of PCR products and not confirmed by sequencing; **Breakpoints inferred by the size of PCR products and not confirmed by sequencing.
