# Supplementary material for: High expression of miR-125b-2 and SNORD116 noncoding RNA clusters characterize ERG-related B cell precursor acute lymphoblastic leukemia
Source: Oncotarget. 2017 Mar 21;8(26):42398–413. doi: 10.18632/oncotarget.16392 (PMC5522075; doi:10.18632/oncotarget.16392)
Supplement: Supplementary file 7 [file oncotarget-08-42398-s007.pdf]

Supplementary table 8: Complete sequence and structure of snoRNAs in the SNORD116 cluster are listed.

Probe set	Transcript Name	Transcript ID	SNOG	Expression	Sequence (5'-C box-ASE 2-C' box-D' box-ASE 1-D box-3')	ASE 1 3'-5'	ASE 2 3'-5'
HBII-85-24_x_st	SNORD116-24-201	ENST00000384549	SNOG2	High in ERG-related group	GGATCGA**TGATGA**CTTTTATACATGCATTCCTTGGAAAG**CTGAACAAAATGAGTG**AAAACTCTATACCGTCATCTTCGTTGAA**CTGA**GGTCC	AAGTTGCTTCTACTGC CATATCTCAAAA	GAAAGGTTCCTTACGTA CATATTTTC
HBII-85-21_x_st	SNORD116-20-201	ENST00000384507	SNOG2	High in ERG-related group	GGATCGA**TGATGA**CTTCCACATATACATTCCTTGGAAAG**CTGAACAAAATGAGTG**AAAACTCTATACCGTCATCCTCGTCGAA**CTGA**GGTCC	AAGCTGCTCCTACTGC CATATCTCAAAA	GAAAGGTTCCTTACATA TACACCTTC
HBII-85-20_x_st	SNORD116-20-202	ENST00000384529	SNOG2	High in ERG-related group	GGATCGA**TGATGA**CTTCCATATATACATTCCTTGGAAAG**CTGAACAAAATGAGTG**AAAACTCTATACTGTCATCCTCGTCGAA**CTGA**GGTCC	AAGCTGCTCCTACTGT CATATCTCAAAA	GAAAGGTTCCTTACATA TATACCTTC
HBII-85-23_x_st	SNORD116-23-201	ENST00000384645	SNOG2	High in ERG-related group	GGATCGA**TGATGA**CCTCAATACATGCATTCCTTGGAAAG**CTGAACAAAATGAGTG**AAAACTCTATACCGTCGTCCTCGTCAAA**CTGA**GGTCC	AAACTGCTCCTGCTGC CATATCTCAAAA	GAAAGGTTCCTTACGTA CATAACTCC
HBII-85-11_st	SNORD116-11-201	ENST00000383882	SNOG2	High in ERG-related group	GGATCAA**TGATGA**CTTCCATACGTGGGTTCCTTGGAAAG**TTGAACAAAATGAGTG**AAAACTTTATACTGTCATCCTCTTCAAA**CTGA**GGTCC	AAACTTCTCCTACTGTC ATATTTCAAAA	TGAAAGGTTCCTTGGGT GCATACCTTC
HBII-85-14_x_st	SNORD116-14-201	ENST00000383894	SNOG2	High in ERG-related group	GGATCGA**TGATGA**CTTCCATATATACATTCCTTGGAAAG**CTGAACAAAATGAGTG**AAAACTCTATACCGTCATTCTCGTCGAA**CTGA**GGTCC	AAGCTGCTCTTACTGC CATATCTCAAAA	GAAAGGTTCCTTACATA TATACCTTC
HBII-85-16_x_st	SNORD116-16-201	ENST00000384533	SNOG2	High in ERG-related group	GGATCGA**TGATGA**CTTTCATACATGCATTCCTTGGAAAG**CTGAACAAAATGAGTG**AAAACTCTATACCGTCATCCTCGTCGAA**CTGA**GGTCC	AAGCTGCTCCTACTGC CATATCTCAAAA	GAAAGGTTCCTTACGTA CATACTTTC
HBII-85-17_x_st	SNORD116-17-201	ENST00000383929	SNOG2	High in ERG-related group	GGATCGA**TGATGA**CTTCCATATATACATTCCTTGGAAAG**CTGAACAAAATGAGTG**AAAACTCTATACCGTCATCCTCGTCGAA**CTGA**GGTCC	AAGCTGCTCCTACTGC CATATCTCAAAA	GAAAGGTTCCTTACATA TATACCTTC
HBII-85-15_x_st	SNORD116-15-201	ENST00000384445	SNOG2	High in ERG-related group	GGATCGA**TGATGA**CTTCCATATATACATTCCTTGGAAAG**CTGAACAAAATGAGTG**AAAACTCTATACCGTCATCCTCGTCAAA**CTGA**GGTCC	AAACTGCTCCTACTGC CATATCTCAAAA	GAAAGGTTCCTTACATA TATACCTTC
HBII-85-22_x_st	SNORD116-22-201	ENST00000384430	SNOG2	High in ERG-related group	GGATCGA**TGATGA**CTTCCATATGTACATTCCTTGGAAAG**CTGAACAAAATGAGTG**AAAACTCTATACCGTCATCCTCGTCGAA**CTGA**GGTCC	AAGCTGCTCCTACTGC CATATCTCAAAA	GAAAGGTTCCTTACATG TATACCTTC
HBII-85-18_x_st	SNORD116-18-201	ENST00000383961	SNOG2	High in ERG-related group	GGATCGA**TGATGA**CTTCCTTATATACATTCCTTGGAAAG**CTGAACAAAATGAGTG**AAAACTCTATACCGTCATCCTCGTCGAA**CTGA**GGTCC	AAGCTGCTCCTACTGC CATATCTCAAAA	GAAAGGTTCCTTACATA TATTCCTTC
HBII-85-27_x_st	SNORD116-27-201	ENST00000516087	SNOG3	High in ERG-related group	GGATCGA**TGATGA**CTTAAAGATTTATCTAATTTAAAT**CTGAACAAAATGAGTG**ACCAAAACACTTCTGTACCACTTCTGTGAG**CTGA**GGTCC	GAGTGTCTTCACCATG TCTTCACAAAACCA	TAAATTTAATCTATTTAG AAATTC
HBII-85-28_st	SNORD116-28-201	ENST00000516123	SNOG3	Variable expression in the two groups	GGATGGA**TGACGA**CTTAAAAATGAATCTCGTTGGAAT**CTGAGCAAAACGAGTG**AGCAAACCACTTCTGTGCAGTTCTGTGAA**CTGA**GGTCA	AAGTGTCTTGACGTGT CTTCACCAAACGA	TAAGGTTGCTCTAAGTA AAAATTC
HBII-85-13_x_st	SNORD116-13-201	ENST00000384408	SNOG2	Variable expression in the two groups	GGACCAA**TGATGA**CTTCCATACATGCATTCCTTGGAAAG**CTGAACAAAATGAGTG**GGAACTCTGTACTATCATCTTAGTTGAA**CTGA**GGTCC	AAGTTGATTCTACTAT CATGTCTCAAGG	GAAAGGTTCCTTACGTA CATACCTTC
HBII-85-8_x_st	SNORD116-8-201	ENST00000384365	SNOG1	Variable expression in the two groups	GGATCGA**TGATGA**GTCCTCCAAAAAAACATTCCTTGGAAAAG**CTGAACAAAATGAGTG**AGAACTCATACCGTCGTTCTCATCGGAA**CTGA**GGTCC	AAGGCTACTCTTGCTG CCATACTCAAGA	GAAAAGGTTCCTTACAA AAAAACCTCCTG
HBII-85-1_x_st	SNORD116-1-201	ENST00000384335	SNOG1	Variable expression in the two groups	GGATCGA**TGATGA**GTCCCCTATAAAAACATTCCTTGGAAAAG**CTGAACAAAATGAGTG**AGAACTCATAACGTCATTCTCATCGGAA**CTGA**GGTCC	AAGGCTACTCTTACTG CAATACTCAAGA	GAAAAGGTTCCTTACAA AAATATCCCCTG
HBII-85-2_x_st	SNORD116-2-201	ENST00000384274	SNOG1	Variable expression in the two groups	GGATCGA**TGATGA**GTCCCCAAAAAAAACATTCCTTGGAAAAG**CTGAACAAAATGAGTG**AAAACTCATACCGTCATTCTCATCGGAA**CTGA**GGTCC	AAGGCTACTCTTACTG CCATACTCAAAA	GAAAAGGTTCCTTACAA AAAAAACCCCTG
HBII-85-29_st	SNORD116-29-201	ENST00000384516	SNOG3	Variable expression in the two groups	GGATCGA**TGATGA**CTTAAAAAAATGGAAACCTTGGAAAT**CTGAACAAAATGAGTG**ACCAAGACACTTCTGTGAG**CTGA**GGTCC	GAGTGTCTTCACAGAA CCA	TAAAGGTTCCAAAGGTA AAAAAATTC
HBII-85-25_s_st	SNORD116-25-201	ENST00000516517	SNOG3	Variable expression in the two groups	GGATCGA**TGATGA**CTTTAAAATGGATCTCATCGGAAT**CTGAACAAAATGAGTG**ACCAAATCACTTCTGTGCCACTTCTGTGAG**CTGA**GGTCC	GAGTGTCTTCACCGTG TCTTCACTAAACCA	TAAGGCTACTCTAGGTA AAATTTC
HBII-85-5_x_st	SNORD116-5-201	ENST00000384462	SNOG1	Variable expression in the two groups	GGATCGA**TGATGA**GTCCCCCATAAAAACATTCCTTGGAAAAG**CTGAACAAAATGAGTG**AGAACTCATACCGTCGTTCTCATCAGAA**CTGA**GGTCC	AAGACTACTCTTGCTG CCATACTCAAGA	GAAAAGGTTCCTTACAA AAATACCCCCTG
HBII-85-3_x_st	SNORD116-3-201	ENST00000384287	SNOG1	Variable expression in the two groups	GGATCGA**TGATGA**GTCCCCCATAAAAACATTCCTTGGAAAAG**CTGAACAAAATGAGTG**AGAACTCATACCGTCGTTCTCATCGGAA**CTGA**GGTCC	AAGGCTACTCTTGCTG CCATACTCAAGA	GAAAAGGTTCCTTACAA AAATACCCCCTG
HBII-85-26_x_st	SNORD116-26-201	ENST00000516006	SNOG3	High in non-ERG-related group	GGATCGA**TGATGA**CTATAAAAAAAAATGGATCTCATCGGAAT**CTGAACAAAATGAGTG**ACCAAATCATTTCTGTGCCACTTCTGTGAG**CTGA**GGTCC	GAGTGTCTTCACCGTG TCTTTACTAAACCA	TAAGGCTACTCTAGGTA AAAAAAATATC
HBII-85-4_x_st	SNORD116-4-201	ENSG00000275529	SNOG1	High in non-ERG-related group	GGATCGA**TGATGA**GTCCCCCCAAAAAAACATTCCTTGGAAAAG**CTGAACAAAATGAGTG**AAAACTCATACCGTCGTTCTCAGCGGAA**CTGA**GGTCC	AAGGCGACTCTTGCTG CCATACTCAAAA	GAAAAGGTTCCTTACAA AAAAACCCCCCTG
HBII-85-10_st	SNORD116-10-201	ENST00000363791	SNOG2	High in non-ERG-related group	AGGTTGA**TGATGA**CTTACATATATACGTTTTTTTTTTTTTTTTGGAAAGG**TGAACAAAATGAGTG**AAAACTCAGTACCATCATCCTCATCTAA**CTGA**GGTCC	AATCTACTCCTACTACC ATGACTCAAAA	GAAAGGTTTTTTTTTTTT TTTTGCATATATACATTC
HBII-85-6_x_st	SNORD116-6-201	ENST00000384711	SNOG1	High in non-ERG-related group	GGATCGA**TGATGA**GTCCTCCAAAAAAAACATTCCTTGGAAAAG**CTGAACAAAATGAGTG**AAAACTCATACCGTCATTCTCATCGGAA**CTGA**GGTCC	AAGGCTACTCTTACTG CCATACTCAAAA	GAAAAGGTTCCTTACAA AAAAAACCTCCTG
HBII-85-12_x_st	SNORD116-12-201	ENST00000384468	SNOG2	High in non-ERG-related group	GGATCAA**TGATGA**CTTCCATATATACATTCCTTGGAAAG**CTGAATAAAATGAATG**AAAACTCTATACCATCATCCTCATTGAA**CTGA**GGTCC	AAGTTACTCCTACTAC CATATCTCAAAA	GAAAGGTTCCTTACATA TATACCTTC

SNORD116 cluster snoRNAs share highly similar structures that include the typical C box (consensus sequence AUGAUGA) near the 5′ end, D box (consensus sequence CUGA) near the 3′end, and the C′ and D′ boxes in the middle of the molecule (consensus sequence CTGAACAAAATGAGTG). Two antisense elements (ASE 1 and ASE 2) are located immediately upstream D and D′ boxes, respectively. In the canonical snoRNAs, ASEs guide the complementarity to target RNA molecule (the residue base paring with the fifth nucleotide upstream D or D′ box will be target of modification). When ASE 1 and ASE 2 of SNORD116 cluster are grouped in antisense direction (3′-5′) and according to the first 10-12 nucleotide sequence upstream D or D′ box, different ASE motifs can be identified (listed in the table). Interestingly, the snoRNAs up-regulated in ERG-related patients share highly similar ASEs motifs.

In the table, snoRNA transcripts name and ID according to Ensembl (http://www.ensembl.org/index.html), snoRNA groups (SNOG) according to Castle et al., 2010 [16].
